# Ginger from Farmyard to Town: Nutritional and Pharmacological Applications

**DOI:** 10.3389/fphar.2021.779352

**Published:** 2021-11-26

**Authors:** Jeremiah Oshiomame Unuofin, Nelisiwe Prenate Masuku, Oluwatomiwa Kehinde Paimo, Sogolo Lucky Lebelo

**Affiliations:** ^1^ Department of Life and Consumer Sciences, University of South Africa, Florida, South Africa; ^2^ Department of Biochemistry, Faculty of Basic Medical Sciences, College of Medicine, University of Ibadan, Ibadan, Nigeria

**Keywords:** ginger, gingerols and shogaols, nutritional composition, *in silico* molecular docking studies, clinical trials, *Zingiber officinale*

## Abstract

Ginger (*Zingiber officinale*) is one of the most widely used natural products consumed as a spice and medicine for treating diabetes, flatulent intestinal colic, indigestion, infertility, inflammation, insomnia, a memory booster, nausea, rheumatism, stomach ache, and urinary tract infections. To date, over 400 bioactive components, such as diarylheptanoids, gingerol analogues, phenylalkanoids, sulfonates, monoterpenoid glycosides, steroids, and terpene compounds have been derived from ginger. Increasing evidence has revealed that ginger possesses a broad range of biological activities, especially protective effects against male infertility, nausea and vomiting, analgesic, anti-diabetic, anti-inflammatory, anti-obesity, and other effects. The pharmacological activities of ginger were mainly attributed to its active phytoconstituents such as 6-gingerol, gingerdiol, gingerol, gingerdione, paradols, shogaols, sesquiterpenes, zingerone, besides other phenolics and flavonoids. In recent years, *in silico* molecular docking studies revealed that gingerol (6-gingerol, 8-gingerol, and 10-gingerol) and Shogaol (6-shogaol, 8-shogaol, 10-shogaol) had the best binding affinities to the receptor protein in disease conditions such as diabetes, inflammation, obesity, and SARS-CoV-2. Furthermore, some clinical trials have indicated that ginger can be consumed for alleviation of nausea and vomiting induced by surgery, pain, diabetes, obesity, inflammation, male infertility. This review provides an updated understanding of the scientific evidence on the development of ginger and its active compounds as health beneficial agents in future clinical trials.

## Introduction

Herbs and spices have been the most sort after throughout history because of the roles they play as food preservatives, flavor, coloring, and therapeutic agents. Although herbs and spices are relatively low-cost commodities, they have been valued as precious jewels by ancient Egypt, India, and China for many centuries (El-Sayed and Youssef, 2019). In recent years, their popularity has increased in developed countries owing to the belief that they are more effective and possess mild adverse effects than synthetic pharmaceuticals for preventing or treating various ailments (Unuofin et al., 2018; Masuku et al., 2020; Unuofin and Lebelo, 2020, 2021). In addition, herbs and spices have gained significant recognition in the food industry due to their incorporation in foodstuffs and their ability to improve health. It has been reported that 70–80% of the world population rely on the use of complementary and alternative traditional medicine (herbal medicine) for major primary healthcare concerns (Chan, 2003; Martín-Domingo et al., 2017; El-Sayed and Youssef, 2019).

According to Herman (2015), herbs are obtained from the leaves of the plant while spices are collected from different parts of the plant such as arils, barks, flowers, fruit berries, pods, rhizomes, roots, and seeds. *Zingiber officinale* (Roscoe) also known as ginger (rhizomes) belongs to the family Zingiberaceae. It is native to East and Southern Asia and consists of 53 genera and 1300 species worldwide, with 80–90 of which are Zingiber (Shahrajabian et al., 2019).

Ginger (*Z. officinale*) is a popular flowering plant whose rhizome, ginger root, or ginger, is widely used as a culinary and folk medicine (Nour et al., 2017). The underground stem (rhizome) is used for the preparation of ginger and can be obtained in colors varying from white to brown, depending on whether the exterior is scraped off and how it is initially treated (Singletary, 2010). The economic importance of ginger has been restricted by challenges such as biotic stress, climatic fluctuations (drought or floods), other external shocks, prominent instability in food price have caused a decline in returns when compared with high production costs (Mmasa and Kizito Mhagama, 2017). According to Mahat et al. (2019), ginger thrives best in a temperature range of 19–28°C, pH (6.0–6.5), and relative humidity (70–90%).

Sowley and Kankam (2019) cited the Transparency Market Research report on ginger, which showed that ginger is among the herbal commodities with great economic value. The top ten ginger-producing countries according to Dhanik and colleagues are India, China, Nepal, Indonesia, Nigeria, Thailand, Bangladesh, Japan, Cameroon, and the Philippines (Dhanik et al., 2017). The global production of ginger in 2017 was estimated to be 3.3 million tonnes. India accounted for 34% of the production while Nigeria, China, and Indonesia produced a substantial amount. Subsequently, China the second-largest producer produced about 0.58 million tons of ginger with a huge amount being exported to Japan, Korea, and Vietnam. In the same year, ginger production witnessed a boost of 6.5% per year in market value and it was projected that by 2022, the consumption rate of ginger would be 7.5% with a valuation as high as US$ 4.18 billion (Dhanik et al., 2017; Sowley and Kankam, 2019; Zhang et al., 2021).

The beneficial effects of ginger to mankind have been dated as far back as the 13th century (Singletary, 2010). The rhizome has been processed into powder, syrup, volatile oil, and oleoresin. The findings of (Ali and Hassan Gilani, 2007) revealed that both the fresh and dried forms of ginger are renowned globally for their culinary and medicinal properties. Literature is replete with information regarding its aromatic, penetrating, spicy, hot, and biting attributes. These attributes, however, are either diminished or lost upon exposure to light and air (Ghayur and Gilani, 2005; Ali and Hassan Gilani, 2007). The use of ginger in Asian and Ayurvedic medicine for the treatment of diabetes, flatulent intestinal colic, indigestion, infertility, inflammation, insomnia, as memory booster, nausea, rheumatism, stomach ache, and urinary tract infections had also been reported (Ali and Hassan Gilani, 2007; Rehman et al., 2010; Kumar Gupta and Sharma, 2014; Mele, 2019). Furthermore, ginger possesses life-improving potential and other pharmacological activities such as anticancer, anti-diabetes antioxidant, antimicrobial, anti-neuroinflammation, chemotherapy-induced nausea, and vomiting (Nile and Park, 2015; Zhu et al., 2018; Crichton et al., 2018; Mao et al., 2019).

Recently the demand and use of ginger and its products (gingerbread, ginger cake, ginger coffee, ginger drink, ginger oil, ginger spice, ginger syrup, and ginger wine) in households, pharmaceutical, brewery, food, and other related industries have skyrocketed (Bag, 2018; Sowley and Kankam, 2019).

Results of recent studies summarized in this review have shown that ginger’s richness in phytochemicals, nutritional potential, economic benefits of cultivation, and export. These studies uncovered the ameliorative effect of ginger against diabetes, obesity, male infertility, and inflammation. Furthermore, the preclinical studies depicting the mechanisms by which ginger elicits its effects are complemented by recent clinical trials that support the traditional view that ginger has analgesic, anti-diabetic, anti-obesity, pain, nausea and vomiting, male infertility, and anti-inflammatory properties. In addition, *in silico* studies (anti-diabetic, anti-obesity, anti-inflammatory, and SARS-CoV-2) have also been reported. This review comprises scientific data on ginger compiled over the past 10 years. These findings strongly support and affirm the widespread belief that ginger’s nutritional and therapeutic properties cannot be downplayed.

## Nutritional and Mineral Composition of Ginger Rhizome From Different Geographical Location

As is the case with many other spices, ginger is rich in proximate composition and mineral elements that are beneficial to the body. The nutritional compositions of ginger grown in Bangladesh, India, Nigeria, and Pakistan are shown in Table 1. with high fluctuations found in the different geographical locations. Indeed, moisture contents varied between 3.7 ± 0.08 and 15.02 ± 0.04%, ash content between 1.74 ± 0.04 and 6.57 ± 0.18%, crude fibers from 0.20 ± 0.05 to 10.36 ± 0.67%, protein contents varied from 4.38 ± 0.3 to 8.58 ± 0.01%, crude fat content ranged from 0.90 ± 0.02 to 11.15 ± 0.00%, and carbohydrates from 38.35 ± 0.1 to 80.3 ± 0.40%.

**TABLE 1:** Comparative data on the nutritional composition of ginger rhizome on a dry weight basis from different geographical locations.

Composition (%)	Dhaka, Bangladesh	Hisar, India	Mysore, India	Enugu, Nigeria	Imo, Nigeria	Kaduna, Nigeria	Kano, Nigeria	Kwara, Nigeria	Port hacourt, Nigeria	Faisalabad, Pakistan
Moisture	7.16 ± 0.04	3.70 ± 0.08	15.02 ± 0.04	6.45 ± 0.00	6.32 ± 0.35	4.61 ± 0.40	4.74 ± 0.30	6.37 ± 0.01	6.67 ± 0.01	8.60 ± 0.23
Ash	3.31 ± 0.12	3.50 ± 0.04	3.85 ± 0.61	6.63 ± 0.00	6.57 ± 0.18	5.25 ± 0.20	5.05 ± 0.10	6.30 ± 0.13	6.40 ± 0.15	1.74 ± 0.04
Crude fiber	4.80 ± 0.12	5.4 ± 0.08	ND	0.92 ± 0.18	10.36 ± 0.67	0.60 ± 0.02	0.20 ± 0.05	3.25 ± 0.13	ND	ND
Crude fat	1.39 ± 0.25	0.90 ± 0.02	3.72 ± 0.03	5.71 ± 0.00	6.48 ± 0.38	7.30 ± 0.50	11.15 ± 0. 00	5.35 ± 0.13	5.53 ± 0.15	5.03 ± 0.43
Crude protein	6.32 ± 0.03	5.80 ± 0.09	5.087 ± 0.09	8.83 ± 0.00	5.45 ± 0.46	4.38 ± 0.30	4.92 ± 0.10	8.58 ± 0.01	8.58 ± 0.01	7.88 ± 0.01
Carbohydrate	77.21 ± 0.22	80.3 ± 0.40	38.35 ± 0.1	71.46 ± 0.00	64.40 ± 0.42	77.86 ± 0.3	73.94 ± 0.20	68.15 ± 0.01	ND	76.4 ± 1.30
Reference	Sarker et al. (2021)	Sangwan et al. (2014)	Shirin Adel and Jamuna, (2010)	Ugwoke and Nzekwe, (2010)	Ogbuewu et al. (2014)	Abubakar et al. (2019)	Abubakar et al. (2019)	Otunola et al. (2010)	Nwinuka et al. (2005)	Shahid and Hussain, (2012)

We observed that ginger grown in Hisar, India exhibited the lowest moisture content (3.7 ± 0.08%) when compared with other studies from different locations(Sangwan et al., 2014). This low moisture level in any food sample is an indicator of its longer shelf life (Unuofin et al., 2017b; Ohikhena et al., 2017). Different reports have shown that low moisture levels of food and agricultural products aid in eliminating the risks of microbial growth and preventing deterioration that may occur during storage (Unuofin et al., 2017b; Ohikhena et al., 2017; Alp and Bulantekin, 2021).

The ash content of ginger gotten from Enugu, Nigeria (Ugwoke and Nzekwe, 2010) had the highest ash content (6.63 ± 0.00%) when compared to other locations as shown in Table 1. It has been revealed that plant materials with high ash content also possess rich dietary fibres content which provides shelter for digestive organisms in the alimentary tract (Schroeder and Bäckhed, 2016; Jimoh et al., 2020). Furthermore, a high level of ash in food and plant material is an indicator of its richness in mineral nutrients (Unuofin et al., 2017a; Ohikhena et al., 2017).

Literature is replete with information regarding the essentiality of fibre in food substances (Unuofin et al., 2017b; Adegbaju et al., 2019; Idris et al., 2019; Abifarin et al., 2021). Our findings revealed that ginger grown in Imo state, Nigeria had the highest crude fibre content (10.36 ± 0.67%) when compared with other geographical locations studied as shown in Table 1. It has been nutritionally established that relatively high dietary fibre composition in food samples is pivotal for proper peristaltic action and aids the absorption of trace elements in the gut and reduces cholesterol absorption (Adebowale et al., 2013; Unuofin et al., 2017a; Ohikhena et al., 2017).

The value of the crude fat of ginger rhizome powder from the different geographical locations ranged from 0.90 ± 0.02 to 11.15 ± 0.00%. It has been suggested that 1–2% of caloric energy from fat is best for healthy living (Antia et al., 2006; Unuofin et al., 2017b). In this regard, the low crude fat content of ginger grown in Hispar, India and Dhaka, Bangladesh (0.90 ± 0.02 and 1.39 ± 0.25%) respectively are within this set caloric energy range when compared with other locations. The onset of several cardiovascular diseases, cancer, and aging have been attributed to excess intake of dietary fat.

On the other hand, the protein contents of ginger grown in Kwara and River States, Nigeria had the highest contents (8.58 ± 0.01%) when compared with other geographical locations as shown in Table 1. Dietary proteins are essential in the human body because they aid in the manufacturing and safeguarding of certain organic materials necessary for their smooth functioning (Hayat et al., 2014).

Carbohydrate had the highest nutritional composition in the ginger derived from all the different locations. However, ginger obtained from Hisar, India (80.3 ± 0.40%) has the highest composition when compared with other geographical locations as revealed in Table 1. The high carbohydrate content makes ginger a rich source of energy and this could be used to enrich the energy content of diets. Ginger is rich in phenolic compounds, i.e., gingerol and shogaol, which have been shown to have molecular structures capable of influencing the thermic effect of food (TEF) (Iwasaki et al., 2006; Fagundes et al., 2021). However, the thermogenic potential of ginger is controversial (Mansour et al., 2012; Gregersen et al., 2013; Fagundes et al., 2021).

Minerals are essential components of the human diet. They support life in the provision of vital nutrients needed for the psychophysical well-being of the body (Unuofin et al., 2017b; Jiménez-Aguilar and Grusak, 2017; Jimoh et al., 2020).

Eleven essential minerals and two heavy metals derived from powdered rhizome samples procured from different geographical locations are compared with reported data (Table 2). The ginger rhizome from the different understudied locations contained beneficial nutrients viz. calcium, potassium, phosphorous, and magnesium in higher concentrations with appreciable amounts of copper, cobalt, iron, nickel, manganese, sodium, and zinc. Calcium, potassium, phosphorous, and magnesium play a crucial role in the bone skeleton, biochemical reactions, and energy metabolism (Haddy et al., 2006; Unuofin et al., 2017b; Srivastava et al., 2019). Furthermore, the data gathered from the different studies revealed that ginger rhizome possesses therapeutic action against growth disorders and anemia due to the availability of iron, manganese, and other mineral antioxidants, e.g., zinc, cobalt, and nickel. The variability in the mineral content of ginger rhizome between different countries, e.g., Bangladesh, China, Ethiopia, India, Nigeria, and Pakistan also within the same country can be observed (Table 2). The variation observed in the nutritional and mineral contents in ginger rhizome could be attributed to the variety used for the study and environmental factors such as climatic conditions, geographical location, genetic and environmental (G×E) interactions like any other plant, soil type, Sun exposure, grazing stress, seasonal changes etc (Hussain et al., 2009; Liu et al., 2016; Srivastava et al., 2019; Unuofin and Lebelo, 2021).

**TABLE 2:** Comparative data on mineral and metal composition of ginger of different geographical location.

Mineral and metals contents in ginger rhizome of different geographical location. Average concentration (mean ± SD, *n* = 3, μg/g dry weight basis)														
Parameter	Dhaka, Bangladesh	China	Tepi, Ethiopia	Bombae, Ethiopia	Hadaro, Ethiopia	Illubabur, Ethiopia	Mysore, India	Imo, Nigeria	Kwara, Nigeria	Kano, Nigeria	Kaduna, Nigeria	Faisalabad, Pakistan	Karachi, Pakistan	Multan, Pakistan
Ca	2080 ± 0.00	2810 ± 0.00	2000 ± 47.00	2540 ± 93.00	2190 ± 24.00	2490 ± 41.00	8840 ± 97.00	34.55 ± 1.39	2800 ± .00	7287.86 ± 0.76	1641.95 ± 0.03	4.9 ± 1.90	ND	ND
Mg	2476 ± 0.00	2762 ± 00.00	2990 ± 9.00	2700 ± 57.00	2760 ± 11.00	4090 ± 105.00	ND	ND	ND	2030.80 ± 1.78	1258.06 ± 0.02	12 ± 14.3	ND	ND
Na	440.19 ± 0.8	474.13 ± 0.6	ND	ND	ND	ND	ND	38.96 ± 3.58	ND	625.00 ± 0.17	333.35 ± 0.76	ND	ND	ND
K	27860 ± 12600	16970 ± 8600	ND	ND	ND	ND	ND	36.34 ± 1.93	ND	429.00 ± 0.76	666.50 ± 0.43	ND	ND	ND
P	ND	ND	ND	ND	ND	ND	17400 ± 120.0	26.70 ± 1.59	8068.0 ± 0.00	3.68 ± 0.02	2.64 ± 0.01	ND	ND	ND
Cu	ND	ND	4.78 ± 0.34	1.86 ± 0.18	2.53 ± 0.19	1.10 ± 0.05	5.45 ± 0.02	0.86 ± 0.01	88.0 ± 0.00	ND	ND	125.0 ± 2.90	1.7 ± 0.01	49.2 ± 2.70
Zn	ND	ND	55.2 ± 3.90	39.6 ± 0.50	38.5 ± 0.50	54.0 ± 2.70	92 ± 0.00	4.19 ± 0.06	640.0 ± 0.00	4.86 ± 0.02	0.99 ± 0.01	122.3 ± 1.60	3.4 ± 0.02	19.7 ± 1.90
Mn	ND	ND	385.00 ± 9.00	285.00 ± 4.30	184.00 ± 3.60	401.00 ± 12.00	913.00 ± 1.00	18.90 ± 1.60	59.0 ± 0.00	20.97 ± 0.07	45.78 ± 0.06	73.30 ± 2.2	1.6 ± 0.01	1014 ± 52.00
Ni	0.558 ± 0.00	ND	5.61 ± 0.44	5.46 ± 0.48	6.78 ± 0.53	8.40 ± 0.32	ND	ND	ND	ND	ND	ND	ND	ND
Fe	195.55 ± 0.00	215.04 ± 0.00	44.2 ± 3.30	55.4 ± 5.00	41.8 ± 2.8	89.0 ± 6.10	800.00 ± 20.0	1.59 ± 0.08	279.74 ± 0.00	39.13 ± 0.05	7.61 ± 0.03	800.00 ± 28.90	13.30 ± 0.01	2475.00 ± 1110.00
Co	0.023 ± 0.00	0.043 ± 0.00	7.58 ± 0.46	5.68 ± 0.40	2.04 ± 0.14	2.18 ± 0.18	ND	ND	ND	ND	ND	ND	ND	ND
Cr	ND	ND	9.28 ± 0.61	6.02 ± 0.14	9.17 ± 0.62	10.80 ± 0.20	7.00 ± 0.00	ND	ND	ND	ND	ND	0.50 ± 0.01	ND
Cd	0.014 ± 0.00	0.042 ± 0.00	0.97 ± 0.08	0.38 ± 0.02	0.38 ± 0.02	0.70 ± 0.07	ND	ND	ND	ND	ND	ND	ND	ND
Reference	Nandi et al. (2013)	Nandi et al. (2013)	Wagesho and Chandravanshi, (2015)	Wagesho and Chandravanshi, (2015)	Wagesho and Chandravanshi, (2015)	Wagesho and Chandravanshi, (2015)	Shirin Adel and Jamuna, (2010)	Ogbuewu et al. (2014)	Latona et al. (2012)	Abubakar et al. (2019)	Abubakar et al. (2019)	Shahid and Hussain, (2012)	Hashmi et al. (2007)	Antia et al. (2004)

The ginger rhizome for the different geographical origins was safe with no critical load of harmful heavy metals. The concentration of metal in plants is largely dependent on its geochemical environment. We observed that there were sufficient essential micronutrients such as Co, Cu, Fe, Mg, Mn, Ni, and Zn (Table 2).

According to Zeiner and Cindrić (2017), it is important to monitor the levels of the heavy metal in medicinal plants and spices used as a nutraceutical/functional food for health-promoting benefits. The levels of heavy metals in medicinal food plants could be increased during postharvest and storage conditions (Srivastava et al., 2019).

## The Phytochemistry of Ginger

Over 400 bioactive components have been found in ginger (Mele, 2019). These chemical constituents have been grouped into different active chemical constituents such as diarylheptanoids, gingerol analogues, phenylalkanoids, sulfonates, monoterpenoid glycosides, steroids, and terpene compounds (Prasad and Tyagi, 2015; Zhang et al., 2021).

### Diarylheptanoids

**FIGURE 1:**
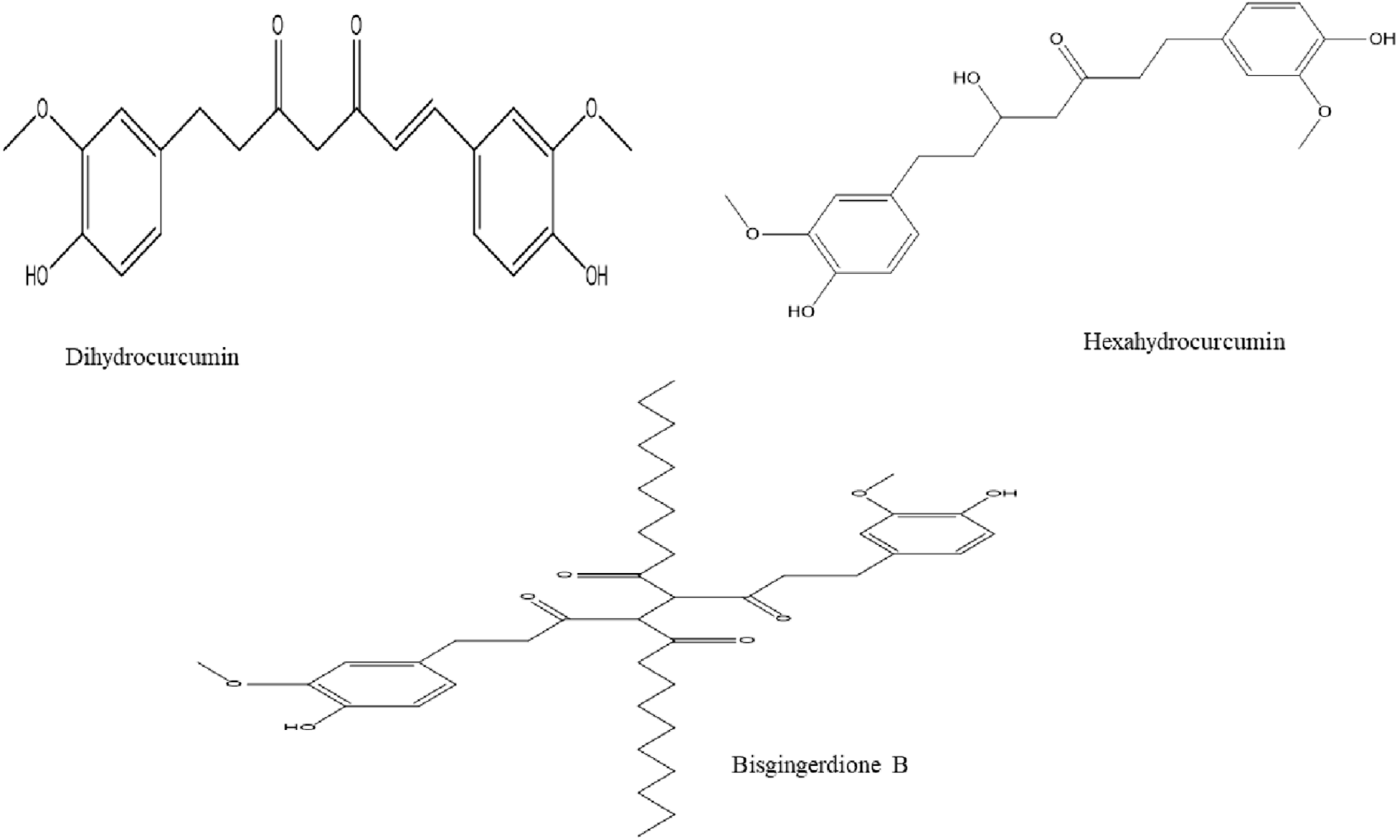
Chemical structures of diarylheptanoids compound isolated from ginger.

Recently, diarylheptanoids with a class term of 1,7-diarylheptane skeleton have gathered an increasing interest (Zhang et al., 2021). Literature is replete with a total of 41 diarylheptanoids compounds derived from ginger e.g. Bisgingerdiones B, Dihydrocurcumin, Hexahydrocurcumin, 6-gingeroldiacetate, and 8-isodehydrogingerdion (Figure 1) (Ma et al., 2004; Jiang et al., 2007; Feng et al., 2011; Hong and Oh, 2012; Olennikov and Kashchenko, 2015). These metabolites possess chemopreventive, anti-hepatotoxic, anti-inflammatory, antioxidant, and anti-tumor (Zhang et al., 2021).

### Gingerol Analogues

**FIGURE 2:**
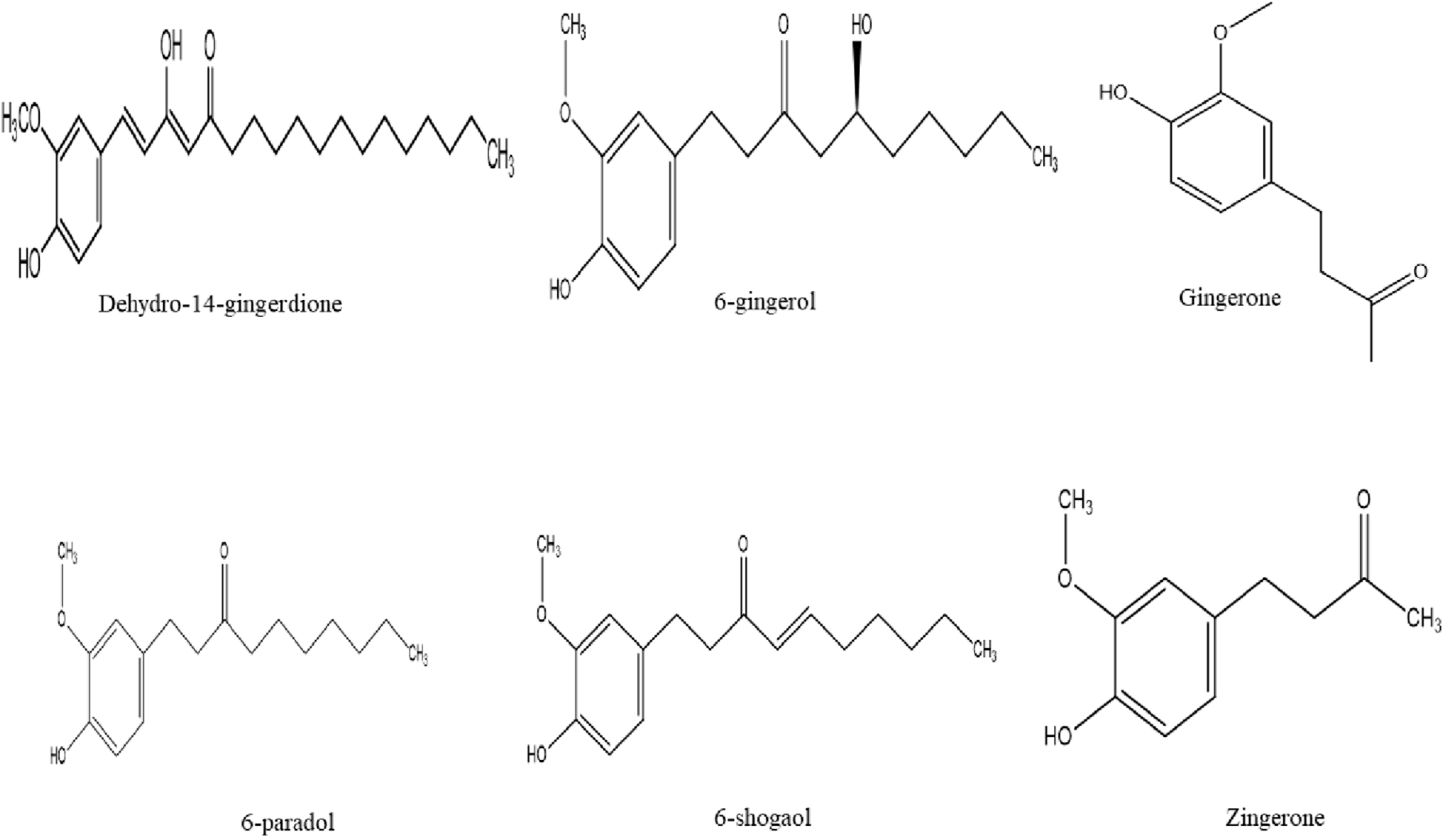
Chemical structures of gingerol analogues compounds isolated from ginger.

Several gingerol analogues such as gingerols, paradols, shogaols, and zingerone possess biting and hot sensations in the mouth and they also exhibit pharmacological effects (Figure 2) (Kubra and Rao, 2012). Gingerols (6-gingerol, 8-gingerol, and 10-gingerol) are the most abundant polyphenol in fresh ginger. Upon dehydration or long-time storage, it can be converted into shogaols which is twice hotter than gingerols. The hydrogenation of shogaols produces paradols (Stoner, 2013). According to Zhang et al. (2021) fresh ginger is not as pungent as dried ginger. In recent years, seventy gingerols analogues compounds have been isolated from ginger such as (5S)-6-gingerol, (5S)-methyl-8-gingerol, (5S)-5-methoxy-6-gingerol, (3R,5S)-6-gingerdiol, dehydro-14-gingerdione, just to mention a few (Jiang et al., 2005; Feng et al., 2011; Lee et al., 2011; Pan et al., 2019).

### Phenylalkanoids and Sulfonates

Six phenylalkanoids compounds (3-hydroxy-1-(4′-hydroxy-3′-methoxy-phenyl)-hexan-5-one, 3-hydroxy-1-(3′,5′-dimethoxy-4′-hydroxyphenyl)-hexan-5-one, 5-hydroxy-1-(4′,5′-dihydroxy-3′-methoxyphenyl)dodecan-3-one, 1-(4′,5′-dihydroxy-3′-methoxyphenyl)dodec-4-en-3-one, (*E*)-3-hydroxy-1-(4′-dihydroxy-3′,5′-dimethoxy-phenyl)-dodecan-6-en-5-one and (E)-3-hydroxy-1-(4-hydroxy-3,5-dimethoxyphenyl)-tetradecan-6-en-5-one) have been reported to present in ginger rhizome (Chen et al., 2011; Chen and Yeh, 2011; Kuo et al., 2012; Li et al., 2013; Wang et al., 2018).

**FIGURE 3:**
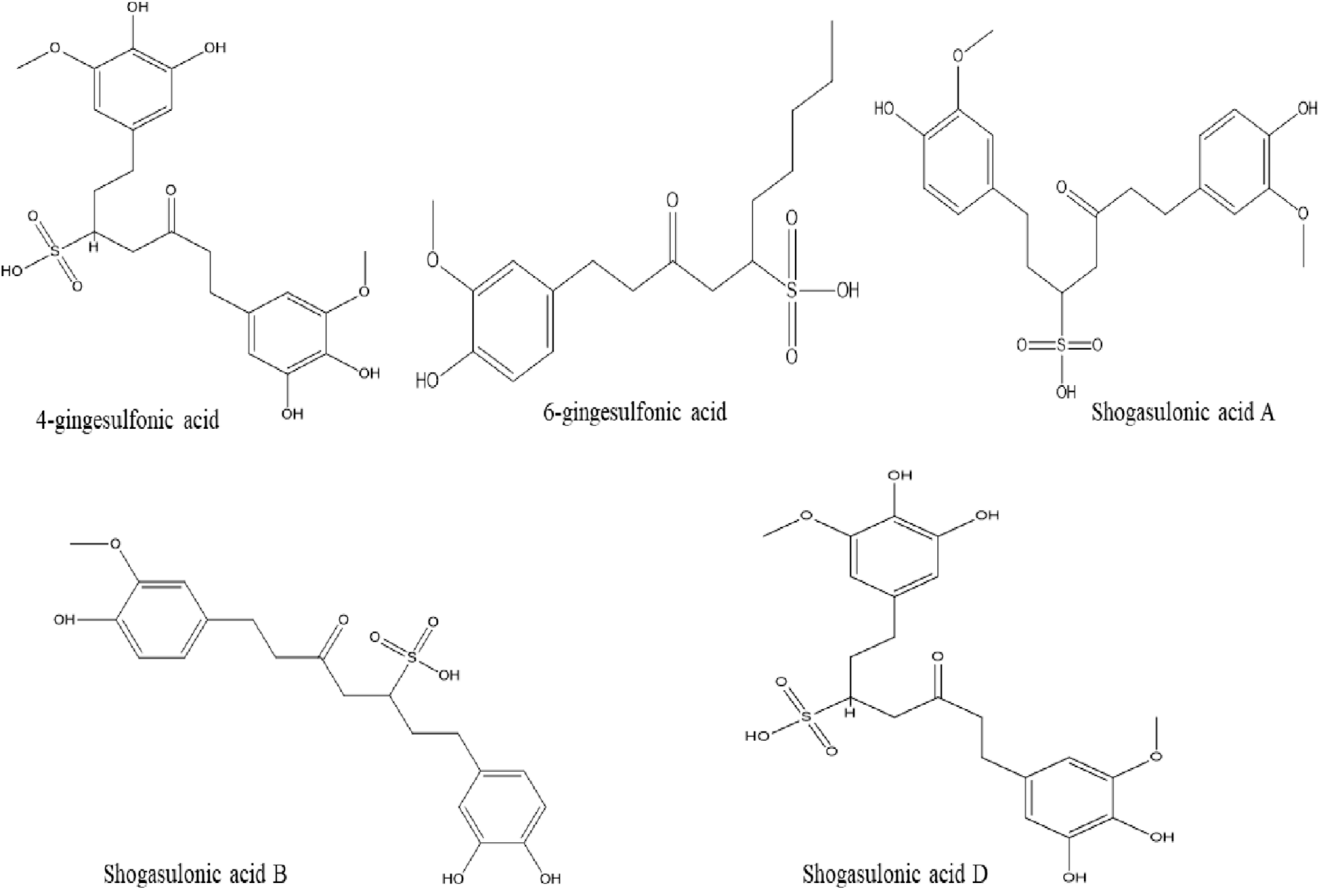
Chemical structures of phenylalkanoids and sulfonates compound isolated from ginger.

Additionally, six sulfonates compounds have been isolated from ginger, they are 4-gingesulfonic acid, 6-gingesulfonic acid, and shogasulfonic acids A-D (Figure 3) (Hori et al., 2003).

### Steroids and Monoterpenoid Glycosides

**FIGURE 4:**
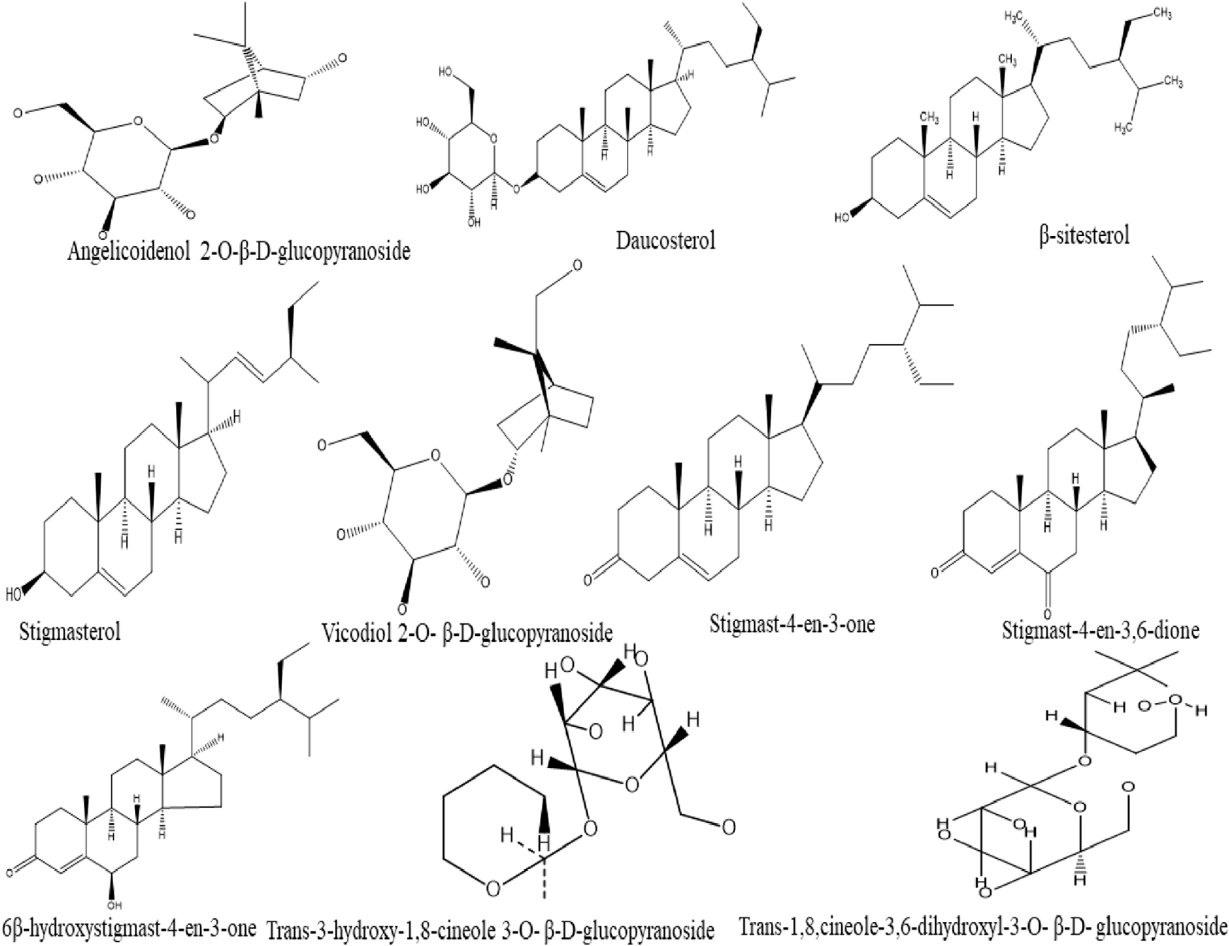
Chemical structures of steroids and monoterpenoid glycosides compounds isolated from ginger.

A total of six steroids compounds including β-sitosterol, daucosterol, stigmast-4-en-3,6-dione, 6β-hydroxystigmast-4-en-3-one, stigmast-4-en-3-one, and stigmasterol have been reported in ginger (Feng et al., 2011). Furthermore, Guo et al. (2018) isolated six monoterpenoid glycosides (Trans-3-hydroxy-1,8-cineole 3,6-dihydroxy 3-O-β-D-glucopyranoside, Trans-3-hydroxy-1,8-cineole3-O-β-D-glucopyranoside,5,9-dihydroxyborneol2-O-β-D-glucopyranoside, Angelicoidenol 2-O-β-D-glucopyranoside, Vicodiol 2-O-β-D-glucopyranoside, and Zingiberoside C) from the fresh rhizome of tongling white ginger (Figure 4).

### Terpenes

**FIGURE 5:**
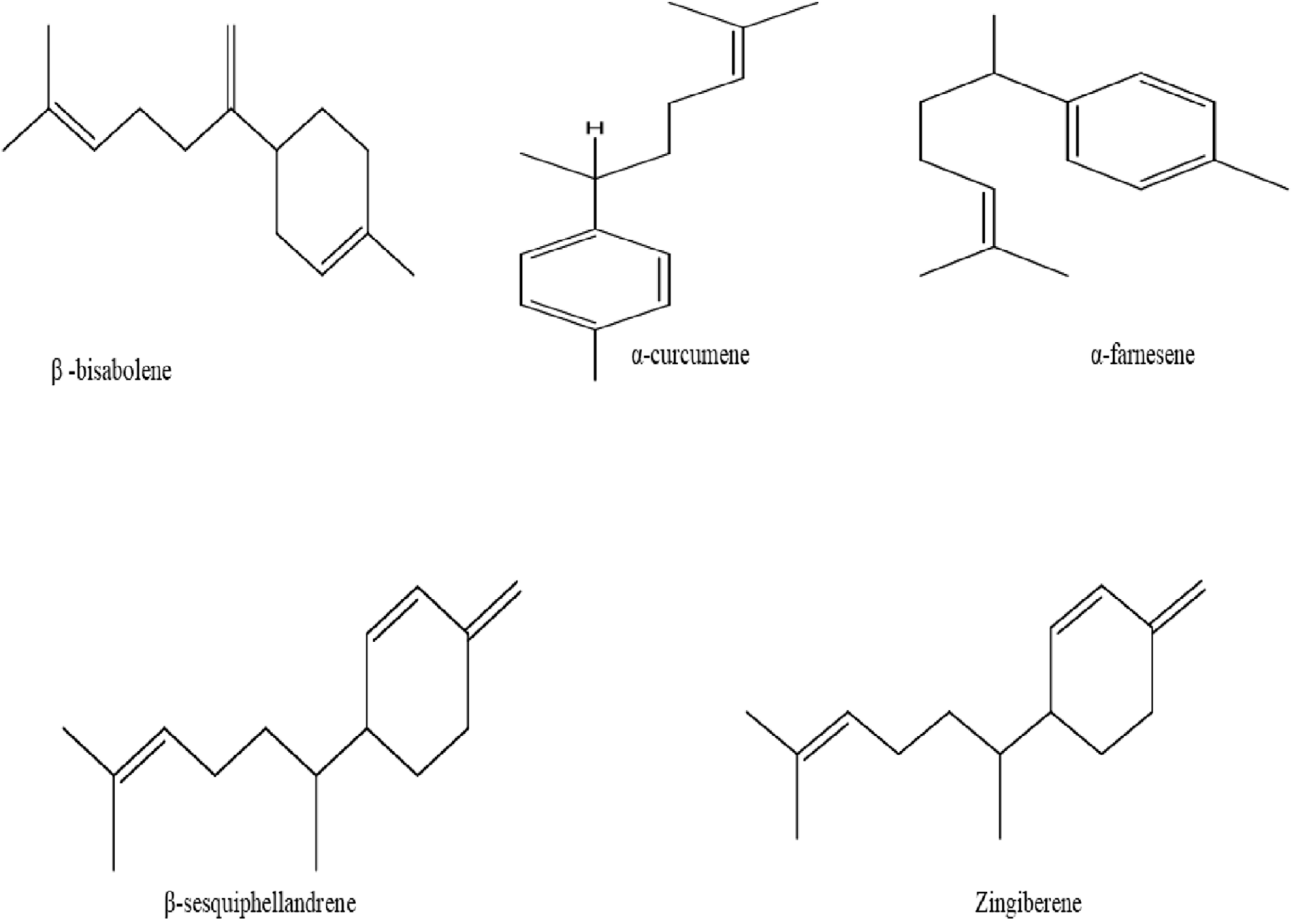
Chemical structure of β-bisabolene, α-curcumene, α-farnesene, β-sesquiphellandrene, and zingiberene isolated from ginger.

Several components of terpenes such as monoterpenes and sesquiterpenes are known to be volatile fractions (Butt and Sultan, 2011). The savory component of ginger is attributed to the presence of sesquiterpenes, while monoterpenes are the most abundant terpenes in fresh ginger oil (Dhanik et al., 2017; Balogun et al., 2019). Furthermore, diverse components present in ginger essential oils are responsible for its aromatic scent, these are such as β-bisabolene, α-curcumene, α-farnesene, β-sesquiphellandrene, and zingiberene (Figure 5) (Prasad and Tyagi, 2015).

## Pharmacological Potential of Ginger and Its Analogues

A large number of bioactive constituents in ginger possess pharmacological activities and they have been comprehensively investigated. These include protective effects against male infertility, nausea and vomiting, analgesic, anti-diabetic, anti-inflammatory, anti-obesity, and other effects. The pharmacological effects of ginger analogous *in vitro* and *in vivo* studies on cells and experimental animals are summarized in Table 3.

**TABLE 3:** Pharmacological effects of ginger analogues.

Activity	Ginger phytocompounds	Dosage used	Mode of action	Experimental Models	References
Anti-emetic	Gingerol	50, 100, and 200 mg/kg, i.g	Inhibits vomiting by attenuating 5-hydroxytryptamine (5-HT) and dopamine (DA) levels in the area postrema and ileum	Cisplatin-induced emesis in adult castrated male minks	Qian et al. (2009)
			Suppresses Substance P (SP) in the mucosa and submucosa of ileum, and neurons of area postrema		
Anti-emetic	Gingerol	50, 100, 200 mg/kg, i.g	Stops vomiting through the inhibition of NK1 receptor in the muscular and submucosa of ileum and the neurons of area postrema, and Substance P receptor in the mucosa and submucosa of the ileum and neurons of the area postrema	Cisplatin-induced emesis in adult castrated male minks	Qian et al. (2010)
Chemotherapy-induced nausea and vomiting (CINV)	Gingerol	20, and 200 mg/kg body weight	Ameliorates gastric emptying through the inhibition of dopamine D2 receptor (D2R) and tyrosine hydroxylase (TH) expression levels and increasing dopamine transporter (DAT)	Cisplatin-induced nausea and vomiting in male Wistar rats	Qian et al. (2016)
Chemotherapy-induced nausea and vomiting (CINV)	Gingerol	10 mg/kg, 20, and 40 mg/kg, i.g in rats	Alleviates chemotherapy-induced nausea and vomiting by reducing the levels of 5-TH, 5-HT3 receptor, TPH, SP, NK1 receptor, PPT, DA, D2R, TH, and boosts accumulation of SERT, NEP, and DAT in the area postrema and ileum	Cisplatin-induced acute and delayed emesis in rats and minks	Tian et al. (2020)
		50, 100, 200 mg/kg, i.g in minks			
Anti-emetic	6-Gingerol	50 and 100 mg/kg	Alleviates vomiting by attenuating 5-hydroxytryptamine (serotonin, 5-HT) concentration through the modulation of tryptophan hydroxylase (TPH), monoamine oxidase A (MAO-A), serotonin reuptake transporter (SERT), and 5-HT3 receptor	Chemotherapy-induced emesis in rats	Cheng et al. (2020)
Anti-obesity	6-Gingerol	50 µmol/L	Suppresses adipogenesis by decreasing the expression of PPARy, C/EBP, mRNA and adipocyte-specific fatty acid binding protein 4 and fatty acid synthase	Rosiglitazone (RGZ)-induced adipogenesis in 3T3-L1 cells	Tzeng et al. (2014)
Anti-obesity	6-Gingerol	6.25, 12.5, and 25 μM	1) Attenuates adipogenesis by suppressing the markers PPARγ, C/EBPα, and adipocyte protein 2, and triglyceride synthesis enzymes, such as sterol regulatory element- binding protein-1, fatty acid synthase, lysophosphatidic acid acyltransferase, and acyl-coA: diacylglycerol acyltransferase	3T3-L1 cells with RAW 264.7 macrophages	Choi et al. (2017)
			2) Reduction in the expression of the proinflammatory cytokines such as TNFα, IL-1β, and IL-6, elevation of cytokine interleukin-10, and inhibition of c-JUN N-terminal kinase (JNK) and I kappa B kinase (IKK)		
			3) Inhibits the induction on nitric oxide synthase (NOS)		
	Gingerol	25, 50 and 75 mg/kg	Cutting down the levels of blood glucose, leptin, insulin, amylase, lipase, and lipids; improve body weight when compared with control group	HFD-induced obese rats	Saravanan et al. (2014)
Anti-obesity	6-Gingerol and 6-Shogaol	0–80 μM	1) Inhibits adipogenesis, induces up-regulation of the brown fat-specific genes expression such as UCP1, PGC1α, PRDM16, Fgf21, Tmem26 and Cidea	Pre-adipocyte cell line (3T3-L1) and Inguinal fat-derived stromal vascular fraction (SVF) cells	Sampath et al. (2021)
			2) Upregulation of mitochondrial biogenesis		
			3) Better binding affinity to the β3-adrenergic receptor membrane protein (β3-AR)		
Anti-obesity	6-shogaol	40 μM	Anti-adipogenic activity by lowering the expression of the PPARγ, C/EBPα, and fatty acid synthase (FAS)	MID-induced adipogenesis in 3T3-L1 Preadipocytes	Suk et al. (2016)
Asthma	Zingerone	5, 20, and 50 μM	1) Increases SOD activity and reduces the MDA levels	MLE12 cells stimulated with hydrogen peroxide (H_2_O_2_)	Zhu et al. (2021)
			2) Suppresses NF-κB activation, reduces mRNA expression of TNF-α and IL-1β		
			3) Inhibit the expression of p65 (nucleus) and p-IκB		
			4) Upregulation of p-AMPK, Nrf2, and HO-1 expression		
Anti-diabetes	6-Gingerol	25 µM	Reduction in level of glucose; enhances cell viability; and inhibition of polyol pathway via decreasing aldose reductase enzyme activity	High glucose-induced human retinal pigment epithelial (HRPE) cells toxicity	Sampath et al. (2016)
Anti-diabetes	6-Gingerol	75 mg/kg	Reduction in the levels of plasma glucose, alanine aminotransferase (ALT), aspartate aminotransferase (AST), advanced glycation end-products (AGEs), and insulin levels	High-fat diet (HFD)-induced high blood glucose in C57BL/6 mice	Sampath et al. (2017)
			Decreasing levels of AGEs and N(ɛ)-(carboxymethyl)lysine (CML) levels through the Nrf2 pathway, increasing GSH/GSSG ratio, heme oxygenase-1 and glyoxalase 1 in liver tissue		
Diabetic nephropathy	Gingerol	12.5, 25, 50, and 100 mg/kg (*in vivo*)	Cutting down the levels of blood glucose, creatinine, and blood urea nitrogen (BUN)	Hf D/STDZ-induced type 2 diabetes in a rat and Normal renal proximal tubular epithelial (NRK 52E) cells treated with high glucose	Song et al. (2019)
		1–50 μM (*in vitro*)	Upsurge in the levels of SOD, GSH, GSH/GSSG ratio, GPx, and CAT.		
			Suppresses activation of NF-κB, renal p38 mitogen-activated protein kinase (p38MAPK) and transforming growth factor-beta (TGF-β); and down-regulation of IL-6, TNF-α, and IL-1β release		
Anti-diabetes	Zingerone	50 and 100 mg/kg body weight	Increases the levels of GSH, SOD, CAT, GPX, and reduces lipid peroxidation	Alloxan-induced diabetic rats	Ahmad et al. (2018)
			Also, decreases the level of NF-kB levels, and down-regulated inflammatory cytokines such as IL1-β, IL-2, IL-6, and TNF-α		
Anti-inflammatory	6-shogaol	20, 30 μM	Inhibition of NF-κB activation, and COX-2 expression by TLR4 pathway	RAW 264.7 cells (a murine monocytic cell line) and 293T human embryonic kidney cells treated lipopolysaccharide (LPS)	Ahn et al. (2009)
			Inhibition of NF-κB activation by MyD88 or IKKβ pathway, and degradation of IRAK-1		
Anti-inflammatory	6-gingerol	50 mg/kg body weight	1) Elevation of hepatic glutathione (GSH), superoxide dismutase (SOD), and glutathione-S-transferase (GST) enzymes, reduction in MDA levels	Diethylenetriamine-induced liver injury in rats	Alsahli et al. (2021)
			2) Restores serum AST, ALT, and ALP, and markedly increases serum total proteins		
			3) Diminishes the expression of inflammatory indicators TNF-α, IL-6, ICAM1, and CRP		
Anti-inflammatory	6-gingerol	0–128 μM	Ameliorates sepsis through the inhibition of pyroptosis and caspase-1p20 release, HMGB1, mature IL-1β, IL-18 by suppressing AMPK activation	ATP and LPS treated RAW264.7 cell line and bone marrow-derived macrophages (BMDMs)	Zhang et al. (2020)
Anti-inflammatory	6-gingerol	2.5, 50, and 100 µM	Suppresses T lymphocyte proliferation through inhibition of DNA synthesis and interferon-γ synthesis, expression of CD25 and CD69 activation markers, cytokine synthesis, and interleukin (IL)-2 receptor signaling	IL-2-dependent mouse CTLL-2 CD8^+^ T lymphocytes	Bernard et al. (2015)
	1-dehydro-10-gingerdione	30 µM	Suppresses NF-kB activation through the inhibition of IkBα phosphorylation by IKKβ	LPS-activated RAW 264.7 macrophages	Lee et al. (2012)
	1-dehydro-10-gingerdione	1–30 µM	1) Attenuates TLR4-mediated expression of NF-kB	LPS-stimulated RAW 264.7 macrophages	Park et al. (2012)
			2) Down-regulation of activator protein 1 (AP-1) target genes, TNF-a and IL-1β, interferon (IFN) regulatory factor 3 (IRF3) target IFN-β gene and IFN-γ inducible protein 10 (IP-10)		
	1-dehydro-10-gingerdione	50, 100, 150, and 200 ng/m	Hinders the production of NO, IL-6, and PGE_2_ via modulating iNOS and COX-2 mRNA expression	LPS-stimulated Raw 264.7 cells	(Han et al., 2013)(Han et al., 2013)
	10-Dehydogingerdione	10 mg/kg	Reduces LDL cholesterol and elevates HDL-cholesterol by suppressing cholesteryl ester transfer protein (CETP)	New Zealand male rabbits fed an atherogenic or high cholesterol diet	Elseweidy et al. (2013)
			Also, decreases cardiovascular risks such as high sensitivity C-reactive protein (hsCRP), oxidized LDL (Ox-LDL), matrix metalloproteinase 9 (MMP9), homocysteine, lipoprotein a (Lp(a))		
	10-Dehydogingerdione	10 mg/kg	Improvement of nuclear factor kappa (NF-kB), insulin-like growth factor I (IGF-I), fibroblast growth factor-23 (FGF-23) of the kidney	Cisplatin-induced nephrotoxicity and renal fibrosis in male Wistar albino rats	Elseweidy et al. (2016)
			Reduces MDA and increases GSH of the kidney		
Anti-neuroinflammatory	12-Dehydogingerdione	2.5, 5, 10 µM	Reduces the production of NO and PGE_2_, and the expression of iNOS, COX-2, and mRNA expression of IL-6	LPS-activated microglial cells and BV-2 cells	Zhao et al. (2019)
			Ameliorates neuro-inflammation by suppressing the Akt/IKK/NF-κB pathway		
			Promotes the production of NO and TNF-α through the activation of NF-E2-related factor (Nrf)-2 and heme oxygenase (Nrf-2/HO-1) pathway		
Anti-inflammatory	Zingerone	25 and 50 mg/kg body weight	1) Attenuates levels of TNF-α, IL-1β, inducible nitric oxide synthase (iNOS), COX-2, p53, cysteine aspartate specific protease-3 (caspase-3), cysteine aspartate specific protease-8 (caspase-8), cytochrome *c*, Bcl-2 associated X protein (Bax), and B-cell lymphoma-2 (Bcl-2)	Vancomycin-induced hepatotoxicity in rats	Kucukler et al. (2020)
			2) Boosting activities of SOD, GPX, and CAT.		
			3) Alleviation of hepatic aspartate aminotransferase, alkaline phosphatase, and alanine aminotransferase		
	Zingerone	50 and 100 mg/kg, p.o	Attenuates accumulation of collagen bundles, TNF-α, and IL-1β levels, MDA level, TGF-β1, and iNOS expression and enhances SOD and GPx activities	Bleomycin-induced pulmonary fibrosis in Wistar-albino rats	Gungor et al. (2020)
	Zingerone	10, 20, and 40 mg/kg	Increases the levels of SOD, GPx, and GSH, and decreases MDA, NO, COX-2, PGE2, TNF-α, and IL-1β	Carrageenan-induced Inflammation in rats	Mehrzadi et al. (2021)
	Zingerone	Orally 25 mg/kg body weight	Improve activities of SOD, catalase and GPx, in the hepatic and joint tissues	Freund’s adjuvant (FCA) immunized arthritic Wistar rats	Bashir et al. (2021)
			Also, reducing levels of NF-κB, TGF-β, TNF-α, IL-1β, IL-6, and Hs-CRP, and induces a significant increase in IL-10 levels		
Nephroprotective	Zingerone	An oral dose of 25 mg/kg body weight	Reduction in levels of malondialdehyde (MDA), nitric oxide (NO) and 8-hydroxy-2-deoxyguanosine in the renal	Adriamycin (doxorubicin)-mediated nephrotoxicity in Swiss albino male mice	Elshopakey et al. (2021)
			Elevation of nuclear factor erythroid 2-related factor 2 mRNA expressions, CAT, SOD, and GSH levels		
			Reduction in the renal levels of NF-κB, TNF-α, IL-1β, and myeloperoxidase activity, thus bringing about anti-inflammation		
Anti-inflammatory	Zingerone	50 mg/kg body weight	1) Reduces the level of MDA, and increases the levels of GSH and CAT, SOD activities	Ovalbumin-induced asthmatic mice	Zhu et al. (2021)
			2) Decreases the level of IL-4, IL-5, IL-13, and increases IFN-γ		
			3) Suppresses the expression of the p-IκB and p65		
			4) Activates the expression of AMPK, Nrf2 (nucleus), and HO-1		
Neuroprotective	6-shogaol and 6-paradol	Oral 5 mg/kg/day, p.o	Diminishing the expression level of TNFα	Experimental autoimmune encephalomyelitis (EAE) C57BL/6 mice	Sapkota et al. (2019)
			Reducing cell accumulation in the white matter of the spinal cord; and also inhibits astrogliosis and microglial activation in the central nervous system		
Gastroprotective	Zingerone	50, 100, and 200 mg/kg, oral	Lowers the level of MDA and restores the NO level	Ethanol-induced gastric ulcers in rat	Karampour et al. (2019)
Anti-inflamma- tory	Zingerone	10, 50, and 100 nM	Stimulates the expression of markers α smooth muscle actin (α-SMA) and smooth muscle 22α (SM22α)), upregulation of AMPK phosphorylation and TIMP4 expression, and reduces the expression of core-binding factor α-1 (CBFA1)	Pi-induced vascular calcification	Lim et al. (2021)
Anti-melanogenesis	8-gingerol	5–100 µM	Suppresses melanogenesis via down-regulation of mitogen-activated protein kinases (MAPK) and protein kinase A (PKA) signaling pathways	B16F10 cells and B16F1 cells (melatonin cells)	Huang et al. (2013)
			Also, reduces microphthalmia-associated transcription factor (MITF) expression and inhibits tyrosinase activity		

## Anti-Diabetes Activities

Ginger has been reported to possess anti-diabetic properties. The aqueous extracts of ginger rhizomes (5, 10, 20, and 40 g/L) were examined on protein glycation and the diffusion of glucose. The results showed that the extract can mitigate diabetes via inhibition of glucose diffusion and by causing a reduction in glycation (Sattar et al., 2012).

A recent study by Ademosun et al. (2021) evaluated the antioxidant properties, glycemic indices, and carbohydrate hydrolysing enzymes activities of ginger-based fruit drinks prepared by mixing ginger (G), pineapple (P), and apple (A) (G50:P40:A10, G50:P30:A20, G50:P20:A30, G50:P10:A40, and G100). The *in vitro* antioxidant activities were assessed using 2,2-diphenyl-1-picrylhydrazyl (DPPH) and 2,2′-azino-bis (3-ethylbenzothiazoline- 6-sulfonic acid) (ABTS) radicals, as well as ferric reducing antioxidant power (FRAP) assays. Results showed that G50:P10:A40 had stronger antioxidant properties against DPPH and ABTS radicals and FRAP compared to other ginger-based drinks and commercial ginger drinks. Also, G50:P10:A40 blend displayed the highest phenolic content and strongest inhibition effect on carbohydrate hydrolyzing enzymes. However, all drinks had low glycemic indices. The study has been suggested that consumption of G50:P10:A40 drinks could be useful in the mitigation of high blood glucose and also the prevention of diabetes mellitus (Ademosun et al., 2021).

Fajrin et al. (2020a) explored the mode of action of ginger extract and 6- shogaol on pancreatic islets and expressions of transient receptor potential vanilloid-1 (TRPV1) and N- methyl-D-aspartate receptor subunit 2B (NMDAR2B) in the spinal cord of streptozotocin (STZ)-induced mice model of Painful Diabetic Neuropathy (PDN). In this study, oral administration of ginger extracts (400 mg/kg body weight), 6-shogaol (15 mg/kg body weight), or gabapentin once daily for 49 days in mice induced a marked decrease of TRPV1 and NMDAR2B expressions in the spinal cord as compared to controls. Also, results revealed that there were no significant differences in the total volume of pancreatic islets and insulin expression between PDN groups. Therefore, the study concluded that ginger extracts and its 6-shogaol compound alleviated pain in PDN by downregulation of TRPV1 and NMDAR2B expressions in the spinal cord, with minor changes on pancreatic islets (Fajrin et al., 2020a).

### Clinical Studies

A double-blind clinical trial study by Javid et al. (2019) evaluated the effects of ginger supplementation on inflammatory, antioxidant, and periodontal parameters in 46 patients with type 2 diabetes mellitus (T2DM) and chronic periodontitis (CP). Treatment with 4 tablets of 500 mg (2 g) of ginger twice per day for 8 weeks with non-surgical periodontal therapy (NSPT) significantly decreased mean levels of tumor necrosis factor-alpha (TNF-α), interleukin-6 (IL-6), pocket depth (PD), hs-C-reactive protein (hs-CRP), clinical attachment loss (CAL) as compared to controls. Also, treatment induced a significant increase in mean serum levels of superoxide dismutase (SOD) and glutathione peroxidase (GPx). It has been suggested that ginger supplementation with NSPT could be recommended for type 2 diabetic patients with CP (Javid et al., 2019).

In another randomized double-blind clinical trial was conducted by (Mohammadzadeh Honarvar and colleagues (2019), 48 diabetic patients were grouped for ginger (2 g) or placebo treatment for 10 weeks. The results demonstrated decreased nuclear factor kappa B (NF-κB) concentration after ginger consumption, but statistically not important. Also, the results showed no significant effect on the anthropometric parameters (hip and waist circumference, and body mass index (BMI) compared to placebo. Owing to insignificant findings, the study suggested that further studies are needed (Mohammadzadeh Honarvar et al., 2019).

In a randomized controlled clinical trial was conducted by Rahimlou et al. (2019), 37 patients with metabolic syndrome (MetS) were randomly allocated to receive ginger powder (2 g) or a placebo for 12 weeks. The application of ginger significantly improved the levels of triglyceride (TG), fasting blood glucose, and insulin resistance as compared to the placebo group. The authors also observed improvements in patients' body weight, waist circumference, total cholesterol level (LDL and HDL), blood pressure, as well as energy intake (Rahimlou et al., 2019).

Recently, a randomized double-blind placebo-controlled clinical trial performed by Hajimoosayi et al. (2020), the determined effect of ginger on the blood glucose level of 70 pregnant women with gestational diabetes mellitus (GDM). They were randomly separated into the ginger group obtained 126 tablets, and the placebo group had 126 tablets for 6 weeks. A significant decrease in fasting blood glucose, fasting insulin, Homeostasis Model Assessment (HOMA) index was observed in the ginger group compared to the placebo group. However, there was no significant reduction in mean blood sugar 2 h post-prandial in both groups (Hajimoosayi et al., 2020).

Another randomized, controlled, and triple-blind clinical trial by Badooei et al. (2021) compared the effects of ginger and aloe vera mouthwashes on xerostomia in type 2 diabetic (TD2) patients. Ginger mouthwash, aloe vera mouthwash, or placebo were used by one-hundred and five (105) patients for 20 ccs three times a day for 14 consecutive days. The study revealed a considerable decreased in all symptoms and severity of xerostomia using ginger and aloe vera mouthwashes. In diabetic patients, a 6.12 ± 2.04 cm decrease of xerostomia was recorded in the ginger group, 4.08 ± 2.09 cm in the aloe vera group when comparing with placebo. The results concluded that ginger mouthwash can effectively ameliorate xerostomia, hence could be prescribed for dry mouth in T2D patients (Badooei et al., 2021).

### *In Silico* Molecular Docking Studies

Research by Fajrin et al. (2018) examined and predicted the binding ability of two ginger constituents to the Transient Receptor potential Vanilloid 1 (TRPV1) using *in silico* molecular docking method. Shogaol (6-shogaol, 8-shogaol, 10-shogaol) and gingerol (6-gingerol, 8-gingerol, and 10-gingerol) as well as were Capsaicin (reference), were docked against TRPV1. The study revealed that 10-gingerol had many hydrogen bonds (six H-bonds), 8-gingerol (5 H-bonds), 8-shogaol, 10-shogaol, 6-gingerol, and 10-gingerol had (four H-bonds), and 6-shogaol (3 H-bonds) bonded to Phe 49 and Ile 293 residues. However, capsaicin had three H-bonds bonded to Phe 54 and Ile 265 of TRPV1. Among the compounds, 6-shogaol also showed potent binding affinity (-7.10 kcal/mol) for TRPV and capsaicin (-7.36 kcal/mol). Because there was no important difference in kcal/mol between 6-shogaol and the drug; therefore, it was noted that 6-shogaol could be developed as TRPV1 for the treatment of Painful Diabetic Neuropathy (PDN) (Fajrin et al., 2018; Fajrin et al., 2020b).

Another study by (Fajrin et al., 2020b) determined the potential activity of 6-paradol and its derivatives to Transient Receptor potential Vanilloid 1 (TRPV1), a target receptor in Painful Diabetic Neuropathy (PDN). In this study, 2-paradol, 4-paradol, 6-paradol, 8-paradol, and 10-paradol were used as potential inhibitors of TRPV1. Capsaicin used in the treatment of PDN was utilized as a reference. The findings demonstrated that 2-paradol, 4-paradol, 6-paradol, 8-paradol, and 10-paradol had a strong binding affinity to the TRPV1. 2-paradol, 4-paradol, and 8-paradol had hydrogen bond interaction with Leu 32 and Thr 28 as capsaicin. 6-paradol had hydrogen bond interaction with Gln 135 and 143 Glu 140. However, 10-paradol had steric interaction with TRV1. It was concluded that 6-paradol and the derivatives potentially inhibited the TRPV1, hence could be used as a drug for PDN therapy (Fajrin et al., 2020a).

**FIGURE 6:**
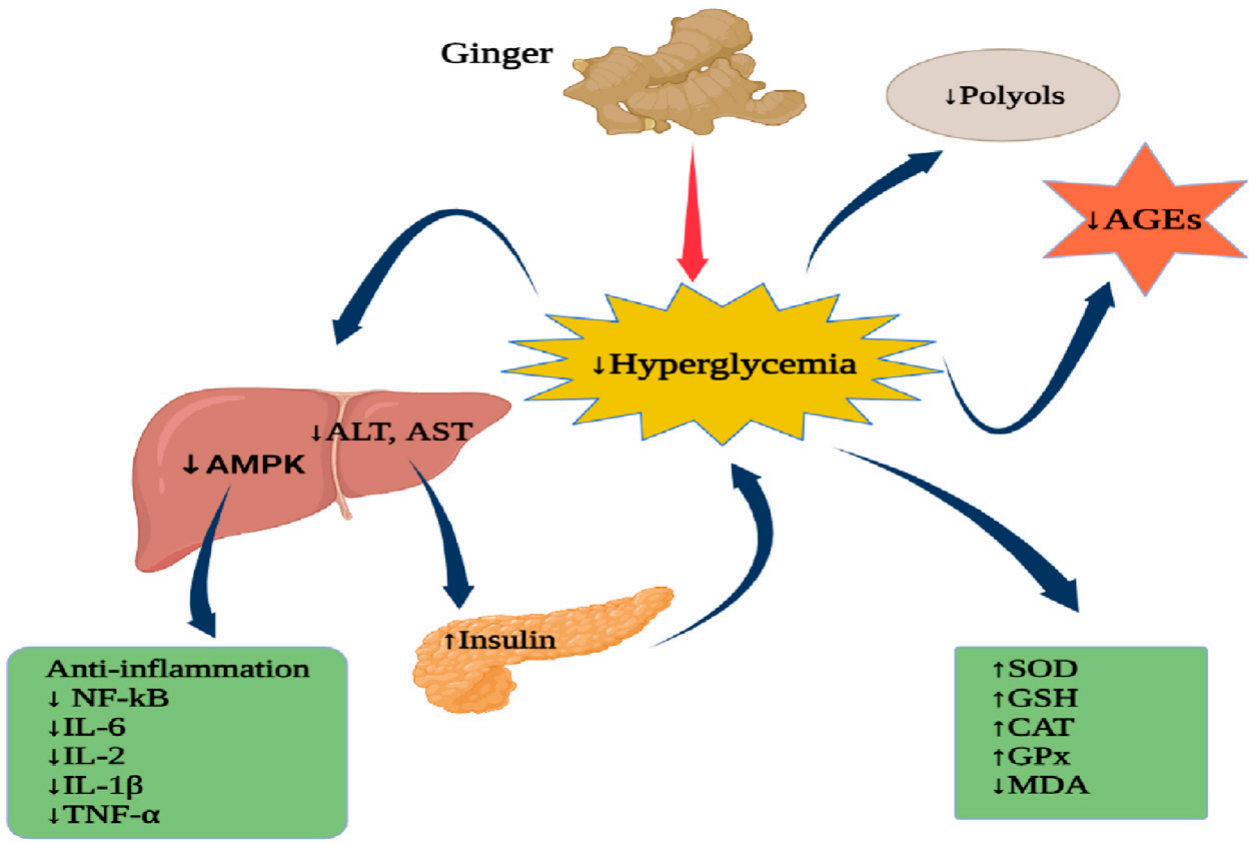
Anti-diabetic actions of ginger. AMPK, Mitogen-activated protein kinase; ALT, Alanine aminotransferase; AST, Aspartate aminotransferase; AGEs, Advanced glycation end-products; NF-kB, nuclear factor kappa B; IL-6, Interleukin-6; IL-2, Interleukin-2; IL-1β, Interleukin-1 beta; TNF-α, Tumour necrosis factor-alpha; SOD, Superoxide dismutase; GSH, glutathione; CAT, Catalase; GPx, Glutathione peroxidase, MDA, Malondialdehyde. Figure was created using BioRender.com by the authors.

Another research examined the potential inhibition activity of ginger compound 6-gingerol on the insulin receptor kinase (3EKK), pancreatic lipase-colipase complex (1N8S), and human alpha-ketoglutarate- dependent dioxygenase FTO (protein 4CXW) for the treatment diabesity. The docking results showed that 6-Gingerol had different binding energy with proteins, 3EKK (−69.79 kcal/mol), IN8S (−53.47 kcal/mol), and 4CXW (−79.33). 6-Gingerol had van der Waal’s interactions with amino residues of 3EKK such as Leu 1002A, Gly 1003A, Gln 1004A, Val 1010A, Ala 1028A, Lys 1030A, Val 1060A, Met 1076A, Met 1139A, Gly 1149A, and Asp 1150A. This compound had also formed hydrophobic interactions with Gly 1003A, Gln 1004A, Gly 1005A, Val 1010A, Ala 1028A, Lys 1030A, Met 1076A, and Met 1139A. On the human pancreatic lipase protein (IN8S), 6-Gingerol formed hydrogen bond interactions with Gln368A andTyr403A amino acid residues. On the human alpha-ketoglutarate-dependent dioxygenase FTO (4CXW) active sites, 6-Gingerol had van der Waal’s interactions with Ile 85A, Pro 93A, Arg 96A, Tyr 108A, Leu 109A, Met 226A, Ala 227A, Val 228A, Ser 229A, His 231A, His 232A, Asp 233A, Glu 234A, and Arg 322A; hydrophobic interactions with Ile 85A, Pro 93A, Leu 109A, Leu 203A, Leu 215A, Val 228A, Ser 229A, and His 231A; aromatic interactions with Tyr 108A and His 231A; hydrogen bonding with Tyr 106A, Glu 234A, and Arg 322A (De et al., 2020). Figure 6 exemplifies the potential anti-diabetic actions of ginger.

## Anti-Emetic Properties

### Clinical Studies

**FIGURE 7:**
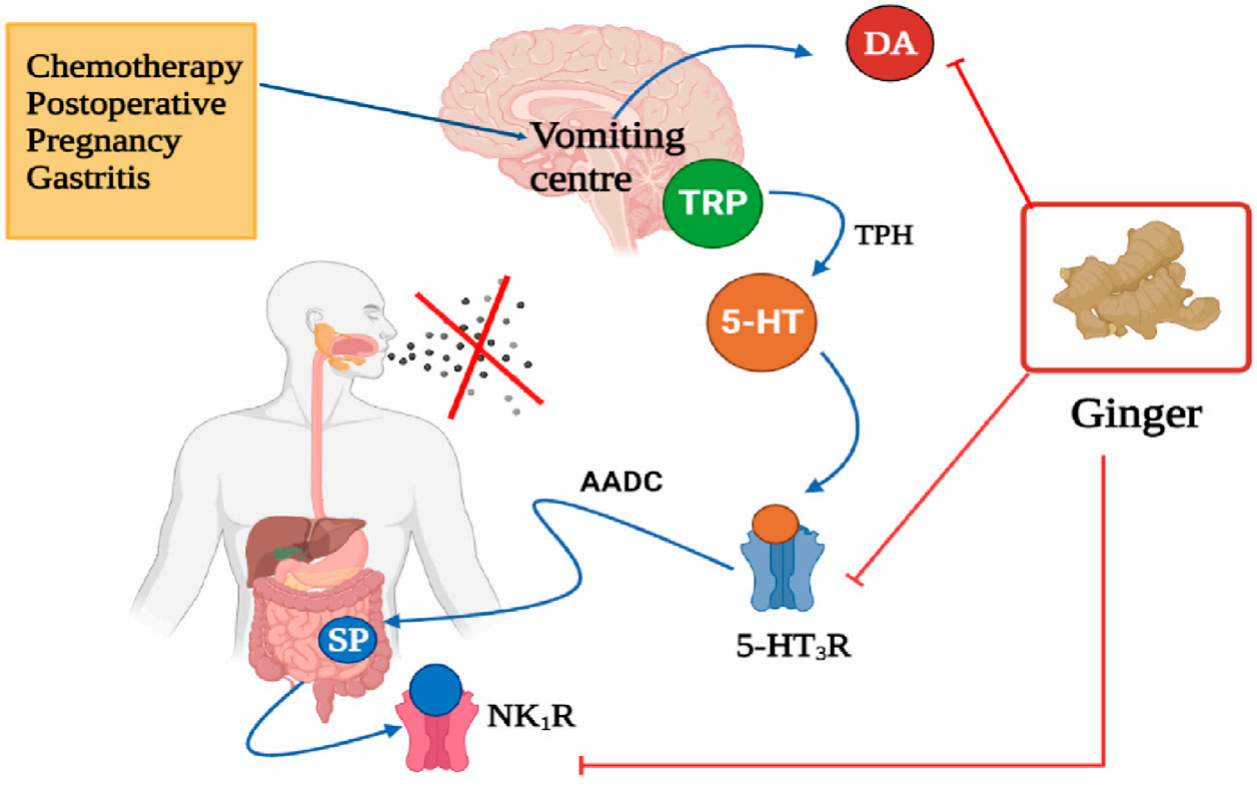
Anti-emetic effect of ginger, targets, and mode of action. TRP, Tryptophan; 5-HT, 5-Hydroxytrptamine (serotonin); 5-HT_3_R, 5-Hydroxytryptamine 3 receptor; AADC, Aromatic L-amino acid decarboxylase; SP, Substance P; NK_1_R, Neurokinin 1 receptor; DA, Dopamine. X represent ceased vomiting. Figure was created using BioRender.com by the authors.

Several studies investigated the utilization of ginger for the alleviation of nausea and vomiting induced by surgery. Kamali et al. (2020) conducted a study that determined the effectiveness of ginger to prevent nausea and vomiting after abdominal hysterectomy as compared to dexmedmoidine (Figure 7). The study involved 92 patients who underwent an abdominal hysterectomy, were randomized to receive orally ginger (1 g) and injection dexmedmoidine (25 mg) before and after the anesthesia. The results demonstrated that ginger was most effective than dexmedetomidine. This study also found that ginger treatment significantly reduced vomiting scores, 2 h after the operation as compared to dexmedetomidine; however, 4 h later both treatments completely stopped vomiting. Also, ginger reduced the number or frequency of nausea than dexmedetomidine (Kamali et al., 2020).

In a double-blind study conducted by Naemi et al. (2020) in 88 patients, age ranges from 30–70 years old (both genders) who were randomly allocated to take various treatments such as ginger (4 capsules), Haloperidol, Metoclopramide, and Dexmedetomidine after laparoscopy found no significant effects on vomiting after the operation (Figure 7). Also, there was no significant difference observed in nausea between treatments. Due to the findings, the study suggested that ginger and these drugs can be further used in the management of vomiting and nausea; however, when there are no serious adverse effects (Naemi et al., 2020). Therefore, patients should be advised to cease using therapy when experiencing side effects.

A triple-blind clinical trial study by Sedighmaroufi and co-workers (2020) assessed the effectiveness of ginger in reducing the frequency and severity of both nausea and vomiting in patients who had eye operations (Figure 7). One hundred and forty-eight (148) patients (all genders) were randomized to obtain treatment with ginger, ondansetron (reference), or placebo. It was found that oral administration of ginger capsules (1000 mg) and ondansetron significantly reduced vomiting as compared to placebo, but no significant difference was observed in the number of vomiting and severity of nausea. They also reported that ginger was more effective, safe, and cheaper as compared to ondansetron, hence be used as an alternative therapy for nausea and vomiting (Sedighmaroufi et al., 2020).

A study carried out by Uthaipaisanwong et al. (2020) examined the efficacy of ginger in 47 gynaecological cancer patients who take combined carboplatin-paclitaxel chemotherapy (Figure 7). As compared to the placebos, dried ginger capsules (500 mg) were showed to be effective in reducing the acute nausea score, but no significant difference was noticed in delayed nausea. The study also observed no significant difference in reducing both acute and delayed vomiting. Besides, heartburn, diarrhoea, and constipation were common adverse effects (Uthaipaisanwong et al., 2020).

A systematic review involved three studies, a pilot, randomized, open-label clinical trial; a randomized controlled trial; and a randomized, double-blind clinical trial was performed to examine the efficacy of ginger on nausea and vomiting associated with chemotherapy (Figure 7). The study found that oral administration of ginger (1–1.5 g) was effective in reducing nausea in patients who had breast cancer chemotherapy, and did not reduce the number of vomiting (Fitriyanti and Sulung, 2020).

Furthermore, the health benefits of ginger in controlling nausea and vomiting during pregnancy have been reported. A study conducted by Anita et al. (2020) evaluated the efficacy of ginger candy in 51 first-trimester pregnant women who experience vomiting three to five times per day (Figure 7). They were grouped (17 each) and recommended to take ginger candy (1/day), vitamin B6 (3 times/day), or a placebo for 7 days. Treatment with ginger candy was effective in reducing the frequency of vomiting (76.5%) as compared to vitamin B6 (5.9%) and placebo (no changes). The study concluded that ginger candy can be used to ameliorate emesis gravidarum or morning sickness (Anita et al., 2020).

A meta-analysis by Hu et al. (2020) conducted on 30 studies or 1174 patients reported that the application of ginger appears to be effective in reducing nausea and vomiting symptoms in pregnant women and nausea as compared to control, but no important effect was detected on vomiting (Figure 7). Besides, intake of ginger showed to be effective than vitamin B6 in the alleviation of nausea and vomiting during gestation (Hu et al., 2020).

## Anti-Inflammatory Activities

The innate immunity, the defense system that protects against pathogens plays a key role in the onset of inflammation *via* germline-encoded pattern-recognition receptors (PRRs). The PRRs such as Toll-like receptors (TLRs), C-type lectin receptors (CLRs), nucleotide-binding oligomerization domain (NOD)-like receptors (NLRs), retinoic-acid-inducible gene-I (RIG-I)-like receptors (RLRs), and receptor for advanced glycation end products (RAGE). The binding of foreign pathogens to PRRs is linked to several signalling pathways such as nuclear factor kappa B (NF-kB), mitogen-activated protein kinase (MAPK), TANK-binding kinase 1 (TBK1)- interferon regulator factor 3 (IRF-3), and the inflammasome, their activation results in the production of proinflammatory factors (Horiguchi et al., 2018; Hu et al., 2018; Li and Wu, 2021).

The increase in levels of inflammatory cytokines such as tumor necrosis factor-alpha (TNF-α), IL-1β, and IL-6, play an essential role in rheumatoid arthritis (Bode and Dong, 2011; Aryaeian et al., 2019). It has been reported that 6- gingerol and 6- shogaol attenuate the production of pro-inflammatory molecules such as prostaglandins by suppressing the enzymes cyclooxygenase (COX)-1 and COX-2 (Grzanna et al., 2005; Saptarini et al., 2013; Mutthuraj et al., 2020).

Several studies demonstrated the anti-inflammatory actions of ginger and its phytocompounds. Zhang et al. (2013) found that 6-gingerol and 6-shogaol, 6-Dehydroshogaol (6-DHSG) (2.5, 5, and 10 µM) were the most powerful inhibitors of inflammation in lipopolysaccharide (LPS)-induced nitric oxide and prostaglandin E_2_ (PGE_2_) production in RAW 264.7 cells. 6-DHSG was powerful in reducing NO and PGE_2_ generation as compared to 6-shogaol and 6-gingerol (Zhang et al., 2013; Mao et al., 2019).

In the study by Tripathi et al. (2008), alcoholic ginger extract (1 µl/ml) caused reduced production of pro-inflammatory cytokines (IL-12, TNF-α, IL-1β) and chemokines (RANTES, MCP-1) on macrophages treated with LPS. The ginger extract decreased the expression of B7.1, B7.2, and MHC class II molecules on macrophages. The study further evaluated the effect of ginger against an antigen and found that ginger extract induced a marked reduction in T cell proliferation as well as production of IFN-γ and IL-2 by T cells (Tripathi et al., 2008).

Luettig et al. (2016) revealed the preventative effect of 6-shogaol pro-inflammatory cytokine tumor necrosis factor α (TNF-α)-induced intestinal barrier inflammation using HT-29/B6 and Caco-2 cells. 6-shaogol prevented upregulation in protein expression of claudin-2 by suppressing phosphatidylinositol-3-kinase/Akt signaling, and dissemble of claudin-1 by inhibition of phosphorylation of nuclear factor kappa light chain enhancer of activated B cells (NF-kB) (Luettig et al., 2016; Mao et al., 2019).

In a recent study, aqueous extract of ginger has been found to possess an anti-inflammatory effect on the paw edema induced by carrageenan 1% of (CAR) injection. Rats treated with an aqueous extract of ginger (100 mg/kg BW) for 1 week before CAR injection showed a marked decrease in edema thickness, size, and percentage of inflammation as compared to indomethacin. The use of ginger extract was more effective, exhibited a considerable anti-inflammatory effect by >80%, at the 5 h after CAR injection. Also, ginger extract normalized the inflammatory markers, fibrinogen, and C-reactive protein (CRP). Besides, this finding was further confirmed by determining the antioxidant activity against inflammation. The SOD, CAT, and GPx activities were significantly higher than the control group (Zammel et al., 2021).

### Clinical Studies

Recently, the study by (Bauer Faria et al., 2021), examined the anti-inflammatory and antimicrobial activity of mouthwash containing 0.5% of ginger essential oil in 31 adult males and females with orthodontic appliances compared with chlorhexidine (0.12%) mouthwash and placebo. The patients were randomized to receive mouthwashes with CLX, ginger, and flavored sterile water (placebo) for 7 days with 15-days intervals between each treatment. Saliva and bleeding were used to assess the efficacy of the mouthwashes. Both ginger and CLX mouthwashes exhibited antimicrobial activity against *Streptococcus mutans*, but different substantivity. Also, ginger mouthwash showed an anti-inflammatory effect, markedly reduced the bleeding as compared to placebo; however, the taste was unpleasant. The study suggested that ginger taste should be improved (Bauer Faria et al., 2021).

A randomized, double-blind, controlled clinical trial conducted by Heidari-Beni et al. (2020) evaluated the efficacy of turmeric extract, black pepper, and ginger formulation on the prostaglandin E_2_ (PGE_2_) in 60 patients with Grade 2 and chronic knee osteoarthritis. The patients were grouped to receive herbal formulation or Naproxen capsule twice a day for 4 weeks. Both oral administration of herbal formulation and Naproxen significantly reduced PGE_2_ levels. The anti-inflammatory effect is associated with gingerol and piperine, some of the known active constituents from ginger and black pepper. It has been concluded that oral intakes of turmeric extract, black pepper, and ginger formulation can ameliorate the PGE_2_ levels in patients with chronic knee osteoarthritis. Therefore, future research examining the anti-inflammatory effect on biomarkers such as ILs and TNF-α is of paramount importance (Heidari-Beni et al., 2020).

Another interesting study by Mutthuraj and co-workers (2020) found that topical application of ginger essential oil on patients suffering from arthritis for 30 days inhibited the pro-inflammatory molecules by reducing the serum levels of rheumatoid arthritis (RA) factor, C reactive protein (CPR), and erythrocyte sedimentation rate (ESR). These results suggest that ginger has pungent anti-inflammatory potential, which can be used in the treatment of joint pain and swelling (Mutthuraj et al., 2020).

A systematic review and meta-analysis by Jalali et al. (2020) assessed the efficacy of ginger on the biomarkers of inflammatory and oxidative stress. The study included clinical studies that determined the effects of ginger on serum CRP (C- reactive protein), TNF-α (tumour necrosis factor-alpha), IL-6 (interleukin-6), PGE2 (prostaglandin E2), TAC (total antioxidant capacity), and MDA (malondialdehyde) until 2019. The effects of ginger on serum CRP, TNF-α, IL-6, TAC, MDA, PGE2 levels were statistically significant. It was concluded that ginger may be used in the treatment or mitigation of inflammation and oxidative stress. Therefore, large-scale randomized clinical trials should be conducted to confirm its safety (Jalali et al., 2020).

A randomized double-blind placebo-controlled clinical trial conducted by Aryaeian et al. (2019) evaluated the effects of ginger on the expression of some immune factors and inflammatory genes in 70 patients with rheumatoid arthritis (RA). The patients were randomized to receive 1500 mg of ginger powder or placebo daily for 12 weeks. The treatment with ginger improved RA through increasing genes expression such as forkhead box P3 (FoxP3), peroxisome proliferator-activated receptor-gamma (PPAR-γ), and GATA binding protein 3(GATA- 3) genes expressions. And downregulation of T-box transcription factor TBX (T-bet), and RAR-related orphan receptor γt (RORγt) genes expression (Aryaeian et al., 2019).

### *In Silico* Molecular Docking Studies

The current study by Zammel et al. (2021) investigated the potential ability of ginger bioactive constituents to bind to the crystal structure of Toll-like receptor 6 (TRL6 4OM7) protein using *in silico* molecular docking. The results showed several ginger phytocompounds were bonded to the TRL6 with different binding affinities ranging between −5.4 and −10.8 kcal/mol. 6-Gingerol had four hydrogen bonds bonded to His651 residue. 8-gingerol showed three hydrogen bonds bonded to Glu 710. 10-Gingerol showed three hydrogen bonds interacting with Tyr 648. 6-Shogaol had five hydrogen bonds linked to Ser 728. Caffeic acid exhibited three hydrogen bonds interacting with Lys 769. Rosmarinic acid and syringic acid formed seven and five hydrogen bonds bonded to His 674 and Ser 728, respectively. Also, amentoflavone and ferulic acid showed five and three hydrogen bonds connected to His 674 and Gln 757 residues. The compounds were further evaluated into human TLR6 and indomethacin, the anti-inflammatory drug was used as a reference. The study found that 6-shogaol was bonded to Ile 684, Asn 687, Glu 675, His 674, Asn 705, Glu 710, and Tyr 648 residues in the pocket region of TLR6. And Rosmarinic acid was interacting with residues Ala 780, Ile 732, Leu 733, Leu 731, Thr 759, His 725, Ser 728, and Gly 727; as compared to indomethacin was bonded to Glu 675, His 651, Glu 650, Glu 710, and Ile 684 (Zammel et al., 2021).

Saptarini et al. (2013), investigated the potential inhibitory effect of gingerol, 6-shogaol, and 6-paradol for anti-inflammation. Molecular docking was studied on COX-1 and COX-2 enzymes. The interaction energy of the compounds toward COX-1 and 2 was ranging from −2.40 to −7.40 kcal/mol, and −7.80 to −11.13 kcal/mol, respectively. The selective index value was further calculated, revealed that all these compounds could induce anti-inflammation vis COX-2. The results of the research suggested that gingerol, 6-shogaol, and 6-paradol should be developed as COX-2 inhibitors for the treatment of inflammation (Saptarini et al., 2013).

Murugesan et al. (2020) examined the potential molecular interactions of nine bioactive constituents of ginger selected from gas chromatography-mass spectrometry (GC-MS) analysis with novel rheumatoid arthritis (RA) target proteins (COX-2, IL-1b, MCSF, MMP-9, and TNF-alpha) for the treatment of rheumatoid arthritis. Ginger methanol extract active compounds included 2,5 dibutylfuran, 6-gingerol, 8-gingingerol, benzoic acid, dihydrocapsaicin, dihydropseudoionone, ferulic acid ethyl ester, geranylacetone, and zingerone. The results indicated different binding affinities toward the proteins, COX-2 (−4.4 to −7.8 kcal/mol), TNF-a (−3.3 to 5.6 kcal/mol, MCSF (−3.6 to 5.7 kcal/mol), IL-1b (−3.2 to 5.7 kcal/mol), and MMP-9 (−4.9 to −7.4 kcal/mol). Amongst nine phytoconstituents, 6-gingerol displayed the best binding affinity with COX-2 and IL-1b (−7.8 and −5.7 kcal/mol, respectively). 8-Gingerol showed a good binding affinity with MCSF (−5.7 kcal/mol) and TNF alpha (−5.6 kcal/mol). Zingerone had a higher binding affinity with MMP-9 protein (−7.4 kcal/mol). 8-Gingerol, 6-gingerol, and zingerone formed the strongest interactions with RA target proteins residues. Moreover, the pharmacokinetic and bioactivity analysis results showed that 6- and 8-Gingerol can act as enzyme inhibitors of Rheumatoid arthritis (RA) proteins (Murugesan et al., 2020).

## Protective Effects Against Male Infertility

Infertility is defined as incompetence to attain pregnancy after a year or more of copulation without contraception. Approximately 50% of men suffer from infertility, 40–90% is attributable to oligospermia and 152 million are associated with erectile dysfunction (Abdillahi and Van Staden, 2012; Masuku et al., 2020, 2021). Several factors that can interfere with fertility include exposure to various chemical compounds, drugs, chronic diseases, and lifestyle factors (Masuku et al., 2020; Kasonga et al., 2021). Numerous studies have investigated the protective and ameliorative properties of ginger male fertility.

Mustafa et al. (2016) investigated the protective effect of ginger on the testicular tissue and testosterone hormone in rats exposed to monosodium glutamate (MSG). The concomitant administration of MSG and ginger aqueous extract (100 mg/kg body weight) for 14 days prevented changes on the stratified epithelium of the seminiferous tubules, spermatogenic cells were normal and well-organized, and normal interstitial space and Leydig cells. The results also showed a significant increase in serum testosterone levels (Mustafa et al., 2016).

Soleimanzadeh et al. (2018) examined the ameliorative effect of ginger against formaldehyde (FA, CH2O)-induced reproductive toxicity in mice. Treatment with FA has been reported to decrease spermatozoa, levels of sex hormones, and antioxidant enzymes activities, and alters the expression of Bcl-2 and Bax genes in the testes. The concomitant administration of FA (10 mg/kg i.p) and ginger ethanolic extract (500, 1000, and 2000 mg/kg/day) in adult male NMRI mice for 35 days demonstrated improvement in spermatozoa parameters, sexual hormones, and antioxidant enzymes. Also, treatment with ginger suppressed the upregulation of Bc1-2 expression and downregulation of Bax gene expression in mice testes. The researchers concluded that the ameliorative effect of ginger was attributed to its androgen, antioxidant, and anti-apoptotic properties. Also, the use of ginger could be useful in patients exposed to FA (Soleimanzadeh et al., 2018).

In another study, oral administration of 70% ethanolic extract of ginger (200 mg/kg/day) for 21 days in aluminum-treated rats induced reduction in activities of liver enzymes (such as AST, ALT, and ALP) and malondialdehyde (MDA). Ginger was found to upsurge the levels of the antioxidants enzymes such as glutathione (GSH), superoxide dismutase (SOD), and catalase (CAT). Also, it was found to enhance the levels of the Follicle-stimulating hormone (FSH), luteinising hormone (LH), and testosterone. Also, the consumption of ginger rejuvenated spermatogenesis. However, coadministration of ginger and taurine was most effective than ginger alone (Kuoti Al-Rekabi, 2019).

Recently, Olusanya et al. (2018) evaluated the protective effect of coadministration of ginger and garlic against *Hibiscus sabdariffa* L. (Roselle)-induced testicular damage in rats. Treatment with a concoction of ginger and garlic aqueous extracts (250 mg/kg) for 28 days displayed an improvement in plasma levels of testosterone, estradiol, prolactin, LH, and FSH. In this study, coadministration ginger plus garlic also preserved the seminiferous tubule structural integrity and prevented the morphological changes of testes. The beneficial effects of ginger are proposed to be associated with its antioxidant properties (Olusanya et al., 2018).

Another recent study by Al-Muswie et al. (2021) examined the effect of ginger aqueous extract on the histological changes of testis and kidneys of male rats treated subjected to hydrogen peroxide (H_2_O_2_). Oral administration of the ginger aqueous extract (0.5 ml of 200 mg/kg) for 30 days attenuated the kidney damage and preserved the testicular damage as manifested by the normal structure of the seminiferous tubules, number, and distribution of spermatogenic cells as well as Leydig cells (Al-Muswie et al., 2021).

Sulfite metabisulfite (SMB) are added to food as preservatives and pharmaceutical agents. SMB impairs spermatogenesis, epidermal morphometry, and spermatozoa parameters in treated rats. Also, SMB decreased levels of enzymatic activities of glutathione peroxidase (GPx), glutathione reductase (GR), and catalase (CAT), and increased malondialdehyde (MDA) of SMB and ginger (500 mg/kg/day) for 28 days preserved histological features of the testes and epididymis and improved levels of enzymatic antioxidants (GPx, CAT, and GR) as well as MDA. They concluded that the prophylactic effects of ginger are related to its androgenic properties and free radicals scavenging capacity (Afkhami Fathabad et al., 2018).

Yaghubi Beklar and co-workers (2019) examined the effect of oral administration of ginger extract (100 mg/kg) on diazinon-induced testicular toxicity in mice. Treatment with ginger for 30 consecutive days caused a significant increase in testosterone levels, inhibitory effect on caspase-3 immunoexpression, and improved the spermatozoa parameters as manifested by a higher percentage of sperm motility and lower sperm abnormality. Also, testicular histopathological examination displayed an improvement. The authors concluded that the protective effects of ginger may be attributed to its anti-apoptotic, antioxidant, and free-radical scavenging activities (Yaghubi Beklar et al., 2019).

The study by Rafiee et al. (2019) assessed the effect of ginger on the zinc oxide nanoparticles (ZNP)-induced spermatogenesis defects in mice. The effects were assessed on the epididymal sperm parameters, testicular histology oxidative stress, serum testosterone level, viability of mouse Leydig (TM3) and mouse Sertoli (TM4) cell lines. Treatment with ginger (10, 20, and 40 mg/kg) before ZNP improved testosterone levels, sperm quality, morphometric parameters, enhanced superoxide dismutase (SOD) and catalase (CAT) activities, and decreased malondialdehyde (MDA). Also, the treatment increased the viability of TM3 and TM4 cells (Rafiee et al., 2019).

Odo et al. (2020) evaluated the preventive effect of ginger extract against lead acetate on sperm quality and haematology in male rats. In this study, the simultaneous ingestion of lead acetate with aqueous ginger extract (300 mg/kg) for 6 weeks induced a significant increase in the spermatozoa parameters (viability and motility), and the production of red blood cells. Also, the level of white blood cells was significantly reduced. The ameliorative impact of ginger on spermatozoa parameters may be associated with its antioxidant properties, and the presence of vitamin C (Odo et al., 2020).

In a study conducted by Mohammed et al. (2019), male rabbit bucks consumed a diet supplemented with fresh ginger (700 g/100 kg) and 6% of *Moringa oleifera* significantly enhanced sexual desire, had higher spermatozoa (count, motility, and morphology) (Mohammed et al., 2019).

A similar study by Adeyemi et al. (2020), assessed the antioxidant properties of ginger on rabbit semen. Consumption of diet ginger (5, 10, and 15 g/kg diet) for 7 weeks by rabbits induced higher semen volume, spermatozoa motility, and seminal total antioxidant capacity especially those fed 15 g of ginger (Adeyemi et al., 2020).

Another study by Kandeil et al. (2019) showed that oral intake of water added ginger extract (100 mg/kg body weight) caused a significant increase in semen volume, and spermatozoa parameters (count, motility, viability), testicular size, production of testosterone, and body weight in V-line male rabbits (Kandeil et al., 2019).

El-Naggar et al. (2020) found that oral intake of ginger extract (400 mg/tablet) before exposure to chronic stress is more effective than after posttreatment. Pre-treatment with ginger improved the gonadosomatic index, testosterone level, and prevented testicular degeneration in male rats (El-Naggar et al., 2020).

The study by Donkor et al. (2018) evaluated the efficacy of ethanolic ginger extract on the semen parameters. Treatment with ginger extract (100 mg/kg, 300 mg/kg, and 500 mg/kg) for 30 days enhanced sperm count, sperm morphology, sperm viability, and sperm motility. The study concluded that could be used in the treatment of male infertility (Donkor et al., 2018).

Muhammad et al. (2019) assessed the effect of ginger (100 mg/kg/day) on reproductive dysfunction in STZ diabetes-induced rats. Treatment with ginger mitigated the inflammation, damage of testicular morphology and also improved spermatozoa quality. The positive effect of ginger on male fertility might be due to its free radical scavenging capacity and anti-inflammatory activity (Muhammad et al., 2019).

The study of Al-Shathly et al. (2020) evaluated the efficacy of ginger in maintaining the structural integrity of testis in streptozotocin (STZ)-induced diabetic rats compared to the efficacy of metformin. Oral administration of aqueous ginger root extracts (500 mg/kg body weight) and metformin for 6 weeks in STZ-induced diabetic male rats resulted in a significant reduction in fasting blood significantly, significantly increased in total antioxidant capacity compared to untreated diabetic rats. Also, treatment with ginger and metformin significantly improved the testicular damage, causes inhibition of caspase-3 immuno-expression, and a significant increase in immune expression of androgen receptors and proliferating cell nuclear antigen. The study concluded that ginger can be given as adjuvant therapy in the treatment of diabetes (Al-Shathly et al., 2020).

Another research evaluated the effects of hydro-alcoholic extract of ginger on HMG-CoA (3-hydroxy-3-methylglutaryl coenzyme A) reductase level in the testis of streptozotocin (STZ)-induced diabetic rats. Oral treatment with hydroalcoholic extract (200 and 400 mg kg/kg) for 56 consecutive days resulted in a significantly upsurged in serum insulin levels, reduction in serum glucose concentration, and HMG-COA reductase level in the rat’s testis compared to the diabetic group. Also, treatment with ginger improved body weight in STZ-induced diabetic rats. The study concluded that the results support the use of ginger in the alleviation of diabetes (Moradi-Podeh et al., 2018).

### Clinical Studies

A few clinical studies have been published that evaluate the use of ginger or phytochemicals for the prevention, alleviation, and or treatment of male infertility.

In 2012, Mares and Najam evaluated the efficacy of taking ginger supplements in 75 infertile men patients aged between 19 and 40 years. In these patients, administration of ginger supplements resulted in a significant increase in spermatozoa parameters (count, motility, viability, and normal morphology), and semen volume compared to before therapy. Also, the use of ginger induced a significant increase in serum glutathione, FSH, LH, and decreased MDA (Mares and Najam, 2012; Gholamnezhad et al., 2018).

A double-blind randomized clinical study by Hosseini et al. (2016) examined the efficacy of ginger on spermatozoa DNA fragmentation in 100 infertile men patients. Patients were randomized to receive 250 mg of ginger powder per capsule and placebo, two times a day for 3 months. Semen samples, before and after treatment were used to assess the spermatozoa count, motility, and DNA fragmentation. Treatment with ginger significantly reduced spermatozoa DNA fragmentation compared to placebo. However, there were no statistically important differences for spermatozoa parameters (count and motility) between the study groups. Besides, no side effects were reported by patients (Hosseini et al., 2016; Gholamnezhad et al., 2018; Banihani, 2019).

## Anti-Obesity Potential

In a mouse model of diet-induced obesity (DIO), consumption of a high-fat diet (HFD, 60% fat w/w) containing ethanolic extract of steamed ginger (SGE) (40 mg/kg and 80 mg/kg) by male C57BL/6J mice for 12 weeks prevented lipid accumulations by suppressing adipogenesis and lipogenesis genes, PPARγ, and C/EBPα expression in 3T3-L1 cells and epididymal adipose tissue of DIO mice. PPARγ and C/EBPα modulate the expressions of aP2, GLUT4, fatty acids synthase (FAS), acetyl-CoA carboxylase (ACC), and adiponectin (ApN). Oral administration of SGE showed a significant reduction in obesity via marked inhibition of aP2, GLUT4, FAS, ACC, and ApN expression. Besides, SGE HFD fed mice exhibited a drastic reduction in total cholesterol triglyceride levels compared to control (Kim et al., 2021).

Concurrently administration of ginger and garlic aqueous extracts (1000 mg/k body weight) in male Wistar rats fed high fat diet (HFD) showed a reduction of body weight and a dose-dependent reduction in the total cholesterol, triacylglycerol (TAG), and low-density lipoprotein (LDL) level (Adegbola et al., 2021).

The study examining the effect of ginger water on body weight and energy expenditure revealed that oral administration of drinking water supplemented ginger water (25 and 50%) in male Wistar rats for a month lowered the total cholesterol and serum triglycerides and significantly reduced the body weight. Also, ginger water attenuated the expressions of mRNA of Sterol regulatory element-binding protein 1 (SREBP-1c) in the liver and leptin in adipose tissues, and increase the expressions of adiponectin, hepatic carnitine palmitoyltransferase1 (CPT-1), acyl-CoA oxidase (ACO), Glucose transporter 2 (GLUT-2), and pyruvate kinase (PK) (Sayed et al., 2020).

In another study, consumption of a high-fat diet supplemented with ginger powder (5%) caused improvements in body weight and gain, hepatic lipid levels, hyperglycemia, hypercholesterolemia, and lipogenic levels in C57BL/6 mice. Also, ginger enhanced levels of the fatty-acid oxidation gene, carnitine palmitoyltransferase 1 (CPT1), and downregulated the adipocyte inflammatory gene expression (Seo et al., 2021).

### Clinical Studies

In a randomized, double-blind, placebo-controlled clinical trial, 80 healthy obese participants were assigned randomly to steam ginger ethanolic extract (SGE) capsules (100 mg plus 5.89–8.83 mg/g of 6-Shogaol) and placebo for 12 weeks. A significant decreased in mean body, body mass index, and body fat level was observed in SGE subjects as compared to placebo. Common cold and dyspepsia were observed in participants after SGE consumption. Nevertheless, the study suggested that SGE and lifestyle modifications together may be useful for maintaining body weight and fat mass (Park et al., 2020).

In another study, Farhadi et al. (2020) assessed the health benefits of aerobic exercise and consumption of ginger extract on lipid profiles, body composition, and liver enzymes in obese menopause women (53–58 years old). Fourth-eighth (48) women were recruited and assigned randomly to the ginger extract (500 mg capsules, 3 times/day) for 24 weeks, aerobic training (3 times/week), and aerobic training -ginger extract, and control. The study found that 12- and 24-weeks of training ginger supplementation, and a combination of ginger and training significantly reduced aspartate aminotransferase (AST) and alanine aminotransferase (ALT) liver enzymes. Also, the combination of ginger and training (12- and 24-weeks) improved lipid profiles and body composition in obese women, and changes were most after 6 months. The results of this study suggested that the combination of aerobic training and ginger extract consumption might be an effective way to improved obesity (Farhadi et al., 2020).

El Gayar et al. (2019) conducted a study on the effect of ginger powder supplementation on glycemic status, lipid profile, and beta-cell function in 80 obese patients with newly diagnosed type 2 diabetes mellitus. The patients were randomized to receive a daily dose of 1.8 g (∼600 mg powered/capsule, 3 times/day) of ginger plus one metformin tablet (850 mg, 2 times/day) with meals for 8 weeks or placebo. The results showed that supplementation with ginger powder resulted in a significant reduction of body mass index, fasting plasma glucose, 2-h postprandial blood glucose, glycated hemoglobin, total cholesterol levels, low-density lipoprotein cholesterol, triglycerides, fasting insulin levels, and homeostasis model assessment-insulin resistance index (HOMA2-IR). The study also found a significant increase in beta-cell function index (HOMA2-%β), insulin sensitivity index (HOMA2-%S), as well as high-density lipoprotein cholesterol levels in the ginger group compared to placebo. The authors concluded that ginger supplements could be used as an adjuvant therapy to improve the efficacy of type 2 diabetes mellitus treatment (El Gayar et al., 2019).

In the study by (Ebrahimzadeh Attari et al., 2016), eighty (80) obese women (18–45 years old) were randomized to receive ginger powered (2 g/day) and placebo for 12 weeks. As compared to the placebo group, oral administration of ginger significantly reduced body mass index, serum insulin, and HOMA-IR index and correlated with increasing quantitative insulin sensitivity check index (QUICKI). Ginger supplementation also significantly reduced serum leptin, resistin, and glucose with both groups, but there was no significant difference. Moreover, there were no significant changes were observed in the body composition and serum levels of adiponectin in both groups. It was concluded that consumption of 2 g of ginger for 12 weeks was not effective enough for weight loss and reducing some of the metabolic features associated with obesity (Ebrahimzadeh Attari et al., 2016; Anh et al., 2020).

**FIGURE 8:**
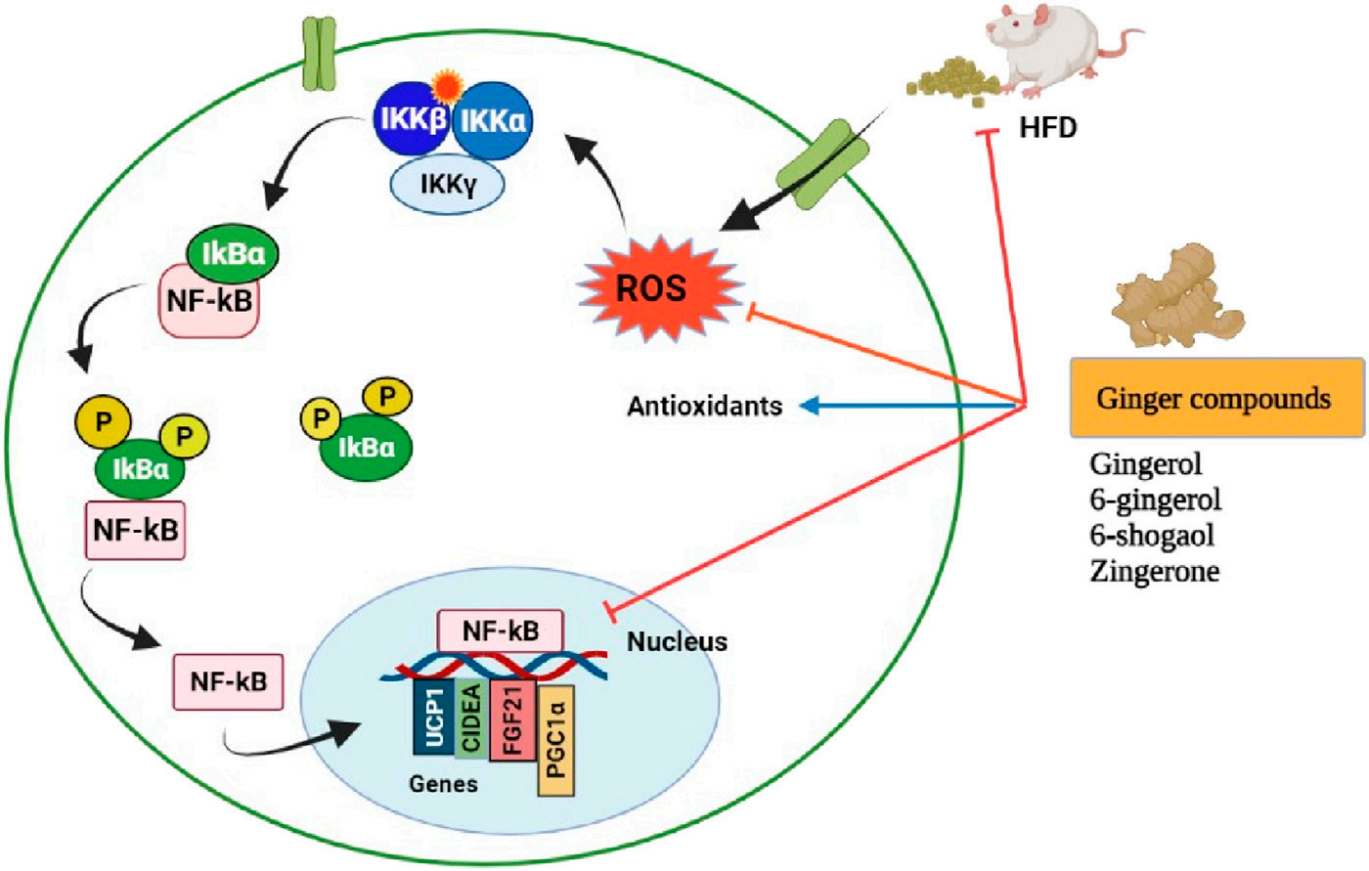
Pharmacological actions of ginger phytoconstituents on obesity. NF-kB, nuclear factor kappa B; IKK (βαγ); I kappa B kinase complex; IkBα, I kappa-B-alpha; UCP1, uncoupling protein 1; CIDEA, Cell death-inducing DFFA like effector A; FGF21, Fibroblast growth factor 21; PGC-1α, Peroxisome proliferator-activated receptor gamma coactivator; ROS, Reactive oxygen species; P, Phosphorylation; HDF, High fat diet. Figure was created using BioRender.com by the authors.

A systematic review with meta-analysis was conducted on 14 randomized controlled trials/473 participants to evaluate the effects of ginger on bodyweight loss, glycemic control, and lipid profiles in overweight and obese individuals. The study revealed that ginger supplementation significantly reduced body weight, waist-to-hip ratio, hip ratio, fasting blood glucose, and insulin resistance index (HOMA-IR), and significantly improved HDL-cholesterol levels. But had no significant effect was found on body mass index (BMI), insulin, triglycerides, and low-density lipoprotein (LDL) (Maharlouei et al., 2019; Venkatakrishnan et al., 2019). The molecular mechanisms for the anti-obesity effects of ginger phytochemicals are illustrated in Figure 8.

### *In Silico* Molecular Docking Studies

The β3-AR has become a promising therapeutic target for the treatment of obesity and other metabolic disorders. This is owing to its presence in the adipose tissue and its ability to activate brown adipocytes which promote energy expenditure, weight loss, improved glucose, and lipid metabolism (Arch and Ainsworth, 1983; Hao et al., 2019). A study conducted by Sampath and co-worker (2021) determined the interactions of 6-gingerol and 6-shogaol as potential ligands for β3-adrenergic receptor membrane protein (β3-AR). They also compared their binding affinity with β3-AR protein by *in silico* molecular docking studies. The study revealed that both 6-gingerol and 6-shogaol had the best binding affinities to the receptor, but 6-gingerol indicated a better −82.364 kcal/mol docking score as compared to 6-shogaol (−79.987 kcal/mol). The study also found that 6-gingerol has powerful hydrogen bond interaction with β3-AR than 6-shogaol. 6-gingerol showed hydrogen bond interaction with serine amino acid residue, and 6-shogaol was bonded to the serine and tryptophan residues. Moreover, the results showed that 6-gingerol enhances the browning effects in white adipocytes via activation of the β3-AR signaling pathway. Therefore, 6-gingerol is a potential treatment for obesity (Sampath et al., 2021).

## Pain-Relieving Potential

### Clinical Studies

A systemic review and meta-analysis of Negi et al. (2021) compared the efficacy of ginger, non-steroidal anti-inflammatory (NSAID), and placebo for primary dysmenorrhea. The application of ginger was found to be effective in the alleviation of menstrual pain as compared to placebo, but no significant difference was observed in the duration of pain. Nevertheless, the use of ginger and non-steroidal anti-inflammatory (NSAID) was useful to treat pain severity (Negi et al., 2021).

A randomized clinical trial by Munir et al. (2021) determined the effectiveness and safety of ginger capsules and naproxen tablets for the treatment of knee osteoarthritis. The study involved 60 male and female patients (>50 years of age), were randomized to receive twice-daily doses of 550 mg of ginger capsules (550 mg) plus 550 mg of naproxen tablets and placebo for 6 weeks. The concomitant administration of ginger with naproxen was more effective than naproxen alone, alleviated pain and stiffness in knee osteoarthritis. Minimal adverse effects were reported. Mild diarrhea was common in patients. In addition, the authors suggested long-term multicenter studies (Munir et al., 2021).

### *In Silico* Study on SARS-Cov-2

Coronaviruses disease 2019 (COVID-19) is a pandemic virus caused by severe acute respiratory syndrome coronavirus 2 (SARS-Cov-2). The virus was first discovered or diagnosed in Wuhan, China by the Chinese Center for Disease Control and Prevention (Guan et al., 2020; Zhu et al., 2020). SARS-Cov-2 is a single-stranded genome (positive sense) made of ribonucleic acid (RNA). The structure of SARS-Cov-2 consists of four main proteins including the membrane (M) glycoprotein, the envelope (E) protein, nucleoprotein (N), and the spike (S) protein (Boheemen et al., 2012; Lu et al., 2020; Tai et al., 2020). The main target of the virus is the angiotensin-converting enzyme 2 (ACE2), an enzyme expressed in several body tissues such as the heart, lungs, testis, and kidneys (Santos et al., 2018; Ni et al., 2020; Fan et al., 2021). The attachment of virus spike protein to the ACE2 receptor on the host cells is the critical step for SARS-Cov-2 entry into cells. After binding to the receptor, the protease enzyme (TMPRSS2) activates the spike protein, and the virus enters the human cells. Once in the cells, the viral replication process takes place (Baughn et al., 2020; Hoffmann et al., 2020; Ren et al., 2020). Fever, dry cough, and feeling tiredness have been reported to be the most common symptoms of COVID-19. Besides, some patients suffer from diarrhea, headache, nasal congestion or runny nose, sore throat, body aches and pains, lymphopenia, and dyspnea (Guan et al., 2020; Orisakwe et al., 2020; Silveira et al., 2020; Wang et al., 2020). The inhibition of SARS-Cov-2 spike protein binding ACE2 using natural herbs or phytochemicals is the promising approach in the prevention and treatment of Covid-19 infection (Abinaya et al., 2020; Mehrotra, 2020; Orisakwe et al., 2020; Silveira et al., 2020; Haridas et al., 2021).

Owing to the urgent need for effective treatment of COVID-19 and fewer treatment options (Ibrahim et al., 2020), ginger extract is prepared by patients as a medication for the management, and melioration of SARS-Cov-2 (Abinaya et al., 2020; Orisakwe et al., 2020). A homemade ginger remedy has been reported. Ginger is prepared alone or in combination with other natural herbs. Ginger, garlic cloves (20), and lime are blended. One tablespoon of paste, Lipton tea and crushed paracetamol tablets (1000 mg) is added to boiling water. One cup of concoction is drunk once after 4 h. The remission of signs and symptoms of COVID-19 has been observed to occur within 3 days. Ginger is cooked with turmeric powder, garlic, and lemon for steam inhalation. The patient covers their head with a towel and deep breath through the nose. The patient recovers from the illness within 3 days. Also, ginger is cooked with garlic, paw leaf, Neem (Dogoyaro) leaf, artemisia, lime, and oranges. Steam is inhaled for 30 min. This is most effective therapy to clear lungs or chest congestion. It takes one to 2 weeks to recuperate (Orisakwe et al., 2020). At the moment, there are no scientific studies and or clinical trials support the efficacy and safety of these medications. Therefore, further studies are needed to prevent their chronic health effects.

*In silico* and molecular docking studies have been conducted to screen the potential inhibition activity of ginger phytochemicals against SARS-Cov-2 and to assess its binding affinities to block SARS-Cov-2. Research by Rajagopal et al. (2020) explored the potential anti-COVID-19 (PD: id-5R82) effectiveness of ginger phytocompounds. The docking results revealed that 8-Gingerol and 10-Gingerol had significant inhibition towards Covid-19. The compounds exhibited higher Glidescore (−5.88 and −5.72 kcal/mol, respectively), high binding affinity to the receptor when compared to that of Hydroxychloroquine drug (−5.47 kcal/mol), and other ginger constituents. Multiple active site residues such Ser 46, Met 49, Hie 41, Gln 189, Arg 189, Asp 187, Met 165, Hie 164, Thr 24, Thr 25, Leu 27, Asn 142, and Gly 143 were involved in the antiviral (5R82) potential of ginger phytochemicals (Rajagopal et al., 2020).

In another study, Haridas et al. (2021) examined 16 compounds isolated from ginger as inhibitors of SARS-Cov-2 spike protein and ACE-2. This study was targeted against COVID-19 protein called SARS-CoV-2 main protease receptor cocrystallized with 6-(ethylamino) pyridine-3-carbonitrile (PDB ID:5R82) using *in silico* molecular docking model. Molecular docking results at the active sites of spike protein two possible inhibitors, adenine and 6-Gingerol showed higher binding affinity (−24.18 and −36.60 kcal/mol, respectively). Adenine formed two hydrogen bonds by interacting with residues Tyr 495, and Gly 496. 6-Gingerol formed four hydrogen bonds with residues Tyr 453, Ser 494, Gly 496, and Tyr 505. Results of the ACE-2 revealed that 6 compounds (10-paradol, 8-paradol, scopoletin, 10-shogaol, 8-gingerol, and 10-gingerol) could be powerful inhibitors of ACE-2 against Covid-19. Docking scores was ranging from −33.72 to −51.27 kcal/mol. 10-Paradol formed two bonds with Arg 273 and Lys 363 residues. 8-paradol formed three bonds with Asn 149 and Lys 363. Scopoletin formed three hydrogen bonds, interacting with residues Asn 149, Asn 368, and Lys 363. 10-shogaol formed three hydrogen bonds, interacting with Asn 277, Lys 363, and Asp 367 residues. 8-gingerol formed three bonds with residues Asp 367 and Lys 363. Also, 10-gingerol formed two hydrogen bonds, interacting with residues Glu 406 and Arg 518 (Haridas et al., 2021).

Moreover, comparative research by Al-Sanea et al. (2021) evaluated the potential inhibition activity of methanolic extract of ginger, ginger silver nanoparticles (AgNPs), strawberry methanolic extract, and strawberry AgNPs to treat SARS-Cov-2. The MTT assay was used to assess the anti-SARS-CoV-2 activity. Ginger AgNPs and strawberry methanolic extract displayed antivirus potentials against Covid-19 with IC_50_ values of 0.034 µg/ml and 0.0062 µg/ml, respectively. Strawberry AgNPs with IC_50_ value of 0.0989 µg/ml and ginger methanolic extract with IC_50_ value of 206.4 mg/ml. To further evaluate the antiviral activity, *in silico* molecular docking study was conducted to examine the potential phytochemicals that might bind to and inhibit SARS-CoV-2 proteins. Among 30 compounds, neohesperidin showed better binding affinity to SARS-CoV-2 NSP16 protein (Al-Sanea et al., 2021).

## Conclusion

Numerous studies have shown that ginger possesses nutritional components required for wellness and its cultivation could boost the economy of many countries especially the developing ones. The presence of superabundant pungent constituents (>400), for instance, gingerols, shogaols, zingerone, and many others provide therapeutic benefits of this plant. In addition, ginger contains high amounts of antioxidants and nutrients which are important in many physiological and biochemical processes in the body. The pharmacological activities of ginger and its chemical compounds are promising in the mitigation, treatment, prevention of diabetes, male infertility, obesity, nausea, emesis, as well as inflammation. Presently, the potential inhibition activity of ginger phytochemicals against SARS-Cov-2 and to assess its binding affinities to block SARS-Cov-2 have been reported. The application of *in silic*o molecular docking tool for the identification of inhibitors or drugs can revolutionize the treatments. The findings of *in silico* molecular studies of ginger compounds interacting with various receptors or proteins (e.g. β3-AR, TRPV1) are promising for the design of novel drugs/inhibitors in combating obesity, diabetes, inflammation, nausea, vomiting, and SARS-CoV-2. However, further studies are needed to investigate their mechanism of action and safety. Moreover, few clinical trials have validated the efficacy and safety of ginger and its constituents especially for the treatment of male fertility. Therefore, future well-designed large-scale randomized clinical investigations of long-term effects are warranted to further evaluate the effectiveness and safety of ginger or phytocompounds for the development of sufficient medications. In addition, owing to the myriad pharmacological properties of ginger, consuming a little bit of ginger in our diet may boost the immune system to fight against various ailments.
